# A meta-analysis of sex differences in human brain structure^☆^

**DOI:** 10.1016/j.neubiorev.2013.12.004

**Published:** 2014-02

**Authors:** Amber N.V. Ruigrok, Gholamreza Salimi-Khorshidi, Meng-Chuan Lai, Simon Baron-Cohen, Michael V. Lombardo, Roger J. Tait, John Suckling

**Affiliations:** a Autism Research Centre, Department of Psychiatry, University of Cambridge, Douglas House, 18B Trumpington Road, Cambridge CB2 8AH, United Kingdom; b Centre for Functional MRI of the Brain (FMRIB), Nuffield Department of Clinical Neurosciences, University of Oxford, John Radcliffe Hospital, Oxford OX3 9DU, United Kingdom; c Department of Psychiatry, College of Medicine, National Taiwan University, No. 1 Jen-Ai Road Section 1, Taipei 10051, Taiwan; d Cambridgeshire and Peterborough NHS Foundation Trust, Elizabeth House, Fulbourn Hospital, Cambridge CB21 5EF, United Kingdom; e Department of Psychology, University of Cyprus, P.O. Box 20537, CY 1678 Nicosia, Cyprus; f Brain Mapping Unit, School of Clinical Medicine, University of Cambridge, Herchel Smith Building, Robinson Way, Cambridge CB2 0SP, United Kingdom; g Behavioural and Clinical Neuroscience Institute, Department of Psychology, University of Cambridge, Downing Street, Cambridge CB2 3EB, United Kingdom

**Keywords:** Brain, Sex differences, Meta-analysis, Gaussian-process regression (GPR), Voxel-based morphometry, Volume

## Abstract

The prevalence, age of onset, and symptomatology of many neuropsychiatric conditions differ between males and females. To understand the causes and consequences of sex differences it is important to establish where they occur in the human brain. We report the first meta-analysis of typical sex differences on global brain volume, a descriptive account of the breakdown of studies of each compartmental volume by six age categories, and whole-brain voxel-wise meta-analyses on brain volume and density. Gaussian-process regression coordinate-based meta-analysis was used to examine sex differences in voxel-based regional volume and density. On average, males have larger total brain volumes than females. Examination of the breakdown of studies providing total volumes by age categories indicated a bias towards the 18–59 year-old category. Regional sex differences in volume and tissue density include the amygdala, hippocampus and insula, areas known to be implicated in sex-biased neuropsychiatric conditions. Together, these results suggest candidate regions for investigating the asymmetric effect that sex has on the developing brain, and for understanding sex-biased neurological and psychiatric conditions.

## Highlights

• This is the first meta-analysis of sex differences in the typical human brain.
• Regional sex differences overlap with areas implicated in psychiatric conditions.
• The amygdala, hippocampus, planum temporale and insula display sex differences.
• On average, males have larger brain volumes than females.
• Most articles providing sex differences in volume are in the ‘mature’ category.

## 1 Introduction

The prevalence, age of onset, and symptomatology of many neurological and psychiatric conditions differ substantially between males and females (Bao and Swaab, 2010; Baron-Cohen et al., 2011; Central Brain Tumour Registry of the United States, 2012; Paus et al., 2008; Rutter et al., 2003). Examples of male-biased conditions include autism, attention deficit/hyperactivity disorder, conduct disorder, specific language impairment, Tourette syndrome, and dyslexia, and examples of female-biased conditions include depression, anxiety disorder, and anorexia nervosa (Bao and Swaab, 2010; Baron-Cohen et al., 2011; Rutter et al., 2003). Factors influencing the asymmetric effect that sex has on brain development may help us understand how and why male and female brains differ in their predisposition for risk for or resilience to such conditions. Identifying where and in what way male and female brains differ will help illuminate these factors and associated mechanisms. Previous whole-brain and region-of-interest studies on sex differences in typically developing human brains show contradictory results, which may be due to small sample sizes and/or variability in age range of the sample in individual studies, leading to opposing or non-significant findings. To summarize the evidence, we report the first meta-analysis of overall and voxel-wise regional brain structure of sex differences in the typically developing human brain and provide a descriptive account of the breakdown of studies providing overall volumes by age category.

Understanding the influence of sex on the developing brain can provide insight into what is happening during the development of psychopathological conditions that are asymmetrically affected between sexes. Sex differences in brain structure are a product of the interaction of biological and environmental influences on brain development (McCarthy and Arnold, 2011). Animal studies have shown that (prenatal) hormones (Arnold and Breedlove, 1985; Phoenix et al., 1959), sex chromosomes (Arnold and Chen, 2009; De Vries et al., 2002), and the immune system (Lenz et al., 2013) all have early roles in the development of neural sexual differentiation. In addition, brain development is also influenced by factors such as sex-biased gene expression (Kang et al., 2011), steroid hormones (Giedd et al., 2012), early life programming such as prenatal nutrition/starvation (DeLong, 1993; Heijmans et al., 2008), stress and maternal infections (Bale et al., 2010), and postnatal factors such as early child care (Center on the Developing Child, 2012; Cicchetti, 2013; Rutter et al., 2003).

Meta-analysis is a statistical framework summarizing themes from the existent literature. Within this framework bias and variability is characterized and quantified leading to a reliable consensus. Recent extension of meta-analysis to brain imaging datasets has identified key regions of structure and function that are consistently detected in a wide range of psychiatric disorders (Etkin and Wager, 2007; Menzies et al., 2008; Valera et al., 2007). However, although a variety of phenomena differ in many psychiatric conditions as a function of sex (Bao and Swaab, 2010; Baron-Cohen et al., 2011; Paus et al., 2008; Rutter et al., 2003), and sex differences in brain function have been systematically reviewed in the typically developing population (Giedd et al., 2012; Sacher et al., 2013; Stevens and Hamann, 2012), to our knowledge no meta-analysis has been conducted on overall or regional voxel-based structural brain differences between human males and females.

In the current study, we carried out two types of meta-analysis. First, we examined sex differences in overall brain volumes. As development and ageing have a large influence on total brain volume, we also investigated if different age categories were well represented in the literature by providing a description of the number of articles, number of total participants and weighted mean volume of each compartmental volume for each of the six age categories. Next, we conducted foci-based meta-analyses on regional differences between males and females, one with voxel-based studies of volume and one with voxel-based studies of tissue density. Gaussian-process regression coordinate-based meta-analysis (GPR-CBMA) was used for the voxel-based meta-analyses, as this new technique allows for relatively more accurate results by incorporating effect-size estimates from source data (Salimi-Khorshidi et al., 2011). Furthermore, GPR-CBMA is also advantageous because its output includes meta-analytic effects in both positive and negative directions as well as an estimate of magnitude models censoring within the source data (i.e., reporting significant foci only), infers the smoothness of meta-analytic statistic images, and provides an effect-size map (i.e., *T*- and/or *Z*-stat) across the entire intra-cranial space.

## 2 Methods

### 2.1 Systematic literature search

The literature search was conducted according to PRISMA guidelines (Moher et al., 2009) for reporting meta-analyses and systematic reviews. The search, conducted in PubMed, Web of Knowledge and Scopus, included articles published between 1990 and January 2013. Search terms used were “brain” AND (sex OR gender OR sex difference OR gender difference) AND (voxel* OR morphometry OR diffusion tensor imaging OR magnetic resonance imaging OR DTI or MRI OR VBM). MeSH terms for “brain” and “sex differences” were also included in the PubMed search.

Authors were contacted if articles were not available online and/or if there was a question about the data presented in the article (e.g., when parameters needed for the meta-analysis, such as effect size information or standard deviations, were not reported in the article). Only articles written in English were included in this analysis. Unpublished materials were not explored and publications performing region-of-interest analysis were excluded. Publications were first selected based on title and then imported into EndNote X4 for abstract selection. After abstract selection, publications were checked for inclusion criteria and reference lists of included articles were crosschecked for potential articles.

### 2.2 Selection criteria

Articles were included in the overall volumes analyses if they explicitly provided (1) any of the following raw (not corrected for age, body size, etc.) mean brain volumes for typically developing males and females: intracranial volume (ICV), total brain volume (TBV), cerebrum (Cb), grey matter (GM), white matter (WM), cerebrospinal fluid (CSF), or cerebellum (Cbl) and (2) standard deviations for these volumes. Articles were included in the regional voxel-based meta-analyses if they provided (1) an explicit whole-brain voxel-based analysis of brain volume or tissue density between typically developing males and females, (2) spatial coordinates for key results, and (3) statistics or effect sizes of key results (*p*, *r*, *F*, *T*, or *Z*-statistics), either present in the publication itself or provided by authors. Cross-sex/gender comparisons in studies performing a patient vs. control analysis were only included if the results of the cross-sex/gender comparison did not spatially overlap with regions showing a sex/gender-by-disorder interaction. All studies included in the analyses were double checked for inclusion criteria by A.N.V.R. and either J.S. or M-C.L.

### 2.3 Data analysis

#### 2.3.1 Overall volumes meta-analysis

In a meta-analysis differences between studies and the omission of studies can bias results. For example, overlooking studies with a negative or non-significant result, perhaps due to publication bias, will tend to overestimate effect sizes. Studies are also likely to have differences in sample populations and study design. This leads to heterogeneity between the studies in the meta-analysis and increases sampling error. Our meta-analyses were therefore tested for bias and heterogeneity of the sample, and based on those outcomes either a random effect model (RFX) or a fixed effect model (FFX) was performed (Higgins et al., 2009). In an FFX it is assumed that there is one true effect size and differences between studies are due to sampling error, whereas in an RFX it is assumed that the true effect may vary from study to study due to differences in their design.

Cochran's *Q* test is the standard test to measure the presence of heterogeneity between studies (Huedo-Medina et al., 2006; Tsoi, 2011). However, the *Q*-statistic does not provide information on the significance of the heterogeneity unlike the *I*^2^ statistic (Huedo-Medina et al., 2006; Tsoi, 2011), which explains how much of the variation between studies in the analysis is due to significant heterogeneity rather than random chance; a meta-analysis with an *I*^2^ of zero means all variability of study effect size estimates is explained by sampling error within studies (Tsoi, 2011). If a significant heterogeneity was found the RFX model was used, otherwise the FFX model was applied.

In order to provide as much detail as possible about the pool of source data that our meta-analysis is based on, forest plots and funnel plots were generated (Salimi-Khorshidi et al., 2009a; Wager et al., 2009). A forest plot reports a summary of the information of individual studies that went into the overall volumes meta-analysis. It is essentially a number of bars with a square in the middle representing the mean effect-size and the length of the bar representing the 95% confidence interval for the mean. They show the amount of variation between the studies and an estimate of the overall result. A funnel plot, on the other hand, is a useful visual aid designed to examine the existence of publication bias (as well as heterogeneity) in systematic reviews and meta-analyses. When plotting the effect-size against its standard error, a symmetric funnel plot implies a ‘well-behaving’ dataset, in which publication bias is unlikely. An asymmetric funnel plot indicates a relationship between effect-size and study size, which may be due to publication bias or small-study effects (i.e., a systematic difference between smaller and larger studies).

#### 2.3.2 Regional coordinate-based meta-analysis

We used Gaussian-process regression coordinate-based meta-analysis (GPR-CBMA), a newly developed tool (Salimi-Khorshidi et al., 2011), to investigate regional sex differences in voxel-based studies of tissue density and volume. In neuroimaging meta-analysis, image-based meta-analysis (IBMA) refers to methods that use full statistic images and allows for the use of hierarchical mixed effects models (accounting for differing intra-study variance and modelling of random inter-study variation). Although IBMA has been shown to be more accurate (Salimi-Khorshidi et al., 2009b), in the absence of full study-level images, CBMA methods have become the standard approach (Eickhoff et al., 2009; Salimi-Khorshidi et al., 2009a). In CBMA, each study included in the meta-analysis is summarized using only the reported (*x*, *y*, *z*) coordinates of peak activations (either with or without activation magnitude). Suppose the full-image study-level data were available, then for study *s* at voxel *k*, the contrast estimates can be modelled as

(1) $$y_{s,k}=\mu_{k}+w_{s,k}\text{,}$$

where $w_{s,k}∼N(0,\sigma_{s,k}^{2}+\tau_{k}^{2})\text{,}$ *μ*_*k*_ denotes the overall population mean (i.e., what a meta-analysis is expected to estimate), *σ*_*s*,*k*_ is within-study standard deviation, *τ*_*k*_ is inter-study standard deviation and $w_{s,k}$ is the observation/reporting error.

Typically CBMA does not have access to study-level *y* and *σ* at every voxel; instead it has access to sparsely sampled standardized effect sizes (i.e., *z* = *y*/*σ*). This changes the model to

(2) $$z_{s,k}=\mu_{k}/\sigma_{s,k}+\sigma_{s,k}\text{,}$$

where $\sigma_{s,k}∼N(0,1+\tau_{k}^{2}/\sigma_{s,k}^{2})$. If we assume that every study has the same *σ* image (i.e., studies are similarly reliable in their effect-size estimates), then the model can be rewritten as

(3) $$z_{s,k}=m_{k}+\sigma_{s,k}\text{,}$$

where $m_{k}=\mu_{k}/\sigma_{k},\;\sigma_{s,k}∼N(0,1+v_{k}^{2})\;\text{and}\;v_{k}^{2}=\tau_{k}^{2}/\sigma_{k})\text{.}$

Even though CBMA only has access to *n* sparsely-located samples of *Z*-stat image (**z** = (**z**_1_, **z**_2_, …, **z**_*n*_)) with their corresponding voxel coordinates **V** = {**v**_1_, **v**_2_, …, **v**_*n*_}, we can employ GPR to model those voxels’ (unobserved) standardized mean effect size *m*. Under GPR, *m* is assumed to be a sample from a Gaussian process, i.e., *m* ∼ *GP*(0, *C*) with **C** denoting the covariance matrix of the GP. We employ a squared exponential (SE) covariance function whose shape can be described with two hyperparameters **σ**_f_ (describing *m*'s variance) and **λ** (describing *m*'s smoothness). Assuming that *z* is sampled from *m* with an additive Gaussian noise of $N(0,\sigma_{k}^{2})$ distribution, results in

(4) $$z_{k}∼N(m_{k},\sigma_{n}^{2})\text{,}$$

in which *σ*_*n*_ estimates $1+\tau_{k}^{2}/\sigma_{s,k}^{2}\text{.}$

In the first step of this solution (inference), the model's hyperparameters (**σ**_*n*_,**σ**_***f***_, and **λ**) are estimated using evidence optimization. These estimates are used in the second step (prediction) to predict the full *m* map. We incorporate our prior knowledge about the smoothness of statistic images by employing a Gamma prior on **λ** in order to minimize the likelihood of an extremely high or low smoothness. This Gamma prior has a shape parameter of 7.7 and a scale parameter of 0.3 (i.e., 90% chance of image's smoothness in FWHM being between 0 and 8 mm).

##### 2.3.2.1 False discovery rate control

Finding the appropriate threshold for voxel-wise meta-analytic statistics can be a challenge. With one test performed for every voxel in the resulting image, some correction of the thresholds is needed to control the overall error rates. Standard procedures for multiple hypotheses testing (e.g., Bonferroni), however, tend to not be sensitive enough to be useful in this context, as they tend to control the chance of *any* false positives (Genovese et al., 2002).

False discovery rate (FDR) controlling procedures, on the other hand, operate simultaneously on all voxel-wise statistics to determine which tests should be considered statistically significant by controlling the expected *proportion* of the rejected hypotheses that are falsely rejected. FDR controlling procedures exert a less stringent control over false discovery compared to family-wise error rate (FWER) procedures, which increases power at the cost of increasing the rate of type I errors. Note that, as the FDR threshold is determined from the observed *P*-value distribution, it is adaptive to the amount of signal in the data (Nichols and Hayasaka, 2003). The *q*-value is defined to be the FDR analogue of the *P*-value. The *q*-value of an individual hypothesis test is the minimum FDR at which the test may be called significant. In this study, *q*-values are estimated for both activation and deactivation images and thresholded in order to control the FDR at voxel level, e.g., at 5%.

## 3 Results

### 3.1 Literature search

The initial search identified 5600 possible articles after duplicates were removed. 5095 articles were excluded after abstract selection because they did not report a sex comparison between typical individuals. An additional 25 articles were found after inspection of the reference lists of the included articles. In total, 167 articles were identified after full-text selection. A total of 126 studies provided total volumes and were included in the overall volumes analysis (see Table 1 for study information and Supplementary Table 1 for an overview of imaging parameters).

Sample overlap in the final study sample was solved by including the studies according to the following weighted criteria: (1) the study with the largest sample (i.e. excluding studies with smaller samples sizes that were part of the same study); (2) the first study using that specific sample (unless a later study included that sample in a larger overall sample); (3) a study that reported a different compartmental volume than a study with the same sample. For example the Sachdev et al. (2008) sample includes the Maller et al. (2006) sample and both are included in the meta-analysis because they report different compartmental volumes. Sachdev et al. (2008) only reports ICV, whereas Maller et al. (2006) reports both ICV and TBV. However, because the Sachdev et al. (2008) sample is larger, the ICV measures of Sachdev et al. (2008) are used in the meta-analysis and the ICV measures of Maller et al. (2006) are excluded. Because Sachdev et al. (2008) does not report TBV, this measure is included from the Maller et al. (2006) paper.

**Fig. 1:**
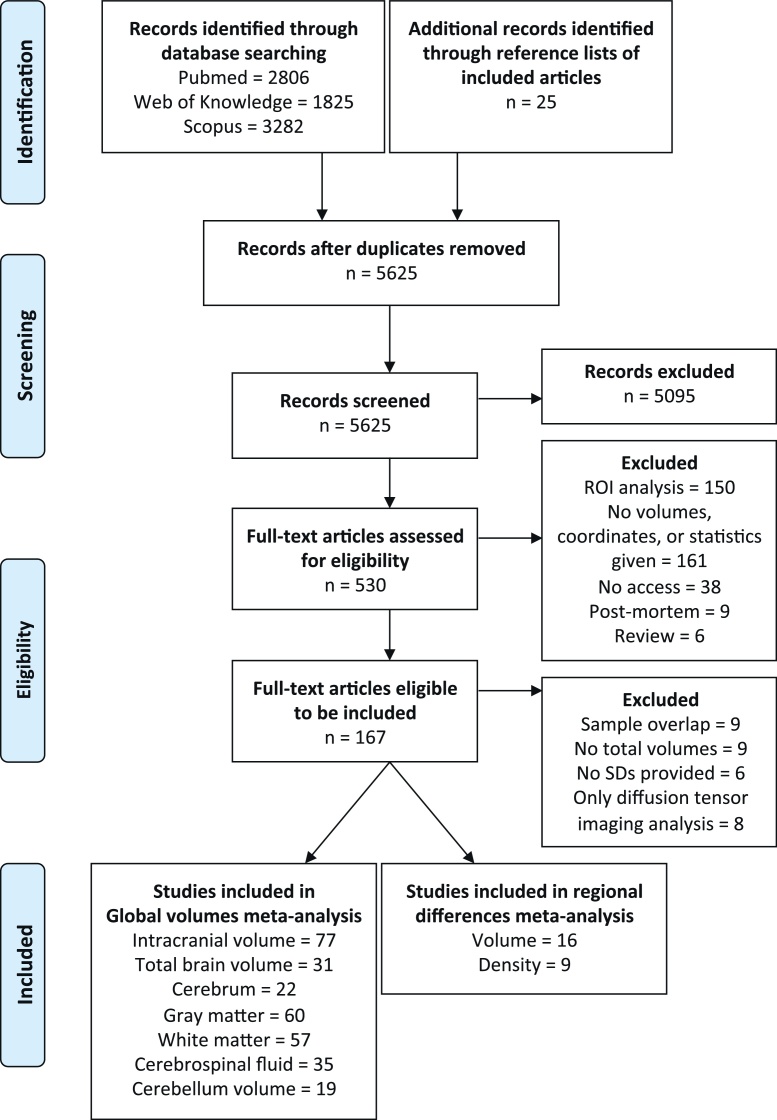
Flow diagram based on PRISMA statement (www.prisma-statement.org).

Articles that performed voxel- or tensor-based morphometry were included in a brain tissue density (9 articles) or brain volume (15 articles) meta-analysis. Another article providing results to a brain volume voxel-based morphometry analysis by our group (Lai et al., 2013) was also included in the voxel-based volume meta-analysis, bringing the total included articles for the volume analysis to 16 (see Table 2 for study demographics and Supplementary Table 2 for an overview of the analysis and imaging parameters). For a complete overview of the data selection, see Fig. 1.

### 3.2 Sex differences in overall volumes

The compartmental brain volumes most often reported in articles include Cbl, CSF, GM, WM, Cb, TBV, and ICV. Separate meta-analyses were conducted for each measurement. Some studies provided total volumes of more than one age- or scanner-matched group, leading to a difference in the number of studies and the number of subject groups in the analyses (see Table 3).

The FFX model was found to be appropriate and hence used for all overall volume meta-analyses except for the TBV analysis, where an RFX model was used. For forest plots of the GM volume meta-analysis, see Fig. 2 and for ICV, TBV, Cb, WM, CSF, and Cbl forest plots see Supplementary Figures 1–6 respectively; for funnel plots see Supplementary Figures 7–13 and Supplementary Methods in the Supplementary Information.

Males have on average larger overall absolute volumes (i.e. not corrected for body size) in each volume category (see Table 3), ranging from 8% to 13% larger volume in males. Sex differences are on average most pronounced in the ICV and Cb volumes. Large effects are also found for TBV, GM, WM, CSF and Cbl volumes.

#### 3.2.1 Breakdown of studies looking at overall volume

Sex differences in total brain volumes vary substantially by chronological age (Brain Development Cooperative Group, 2012; Koolschijn and Crone, 2013; Lenroot et al., 2007; Li et al., 2014; Pfefferbaum et al., 2013). Many study samples in the present meta-analysis cover a large age range: some span from birth to 18 years old or 18–60 years old, whilst others include ages from 1 to 80 years old. Only some report sex differences in compartmental volumes separately for different age groups. Unfortunately, not all studies included here reported information on sex-by-age interactions so this could not be meta-analytically investigated. As an alternative, we wanted to examine the average compartmental volumes change across age ranges as a descriptive report of any chronological age effect. However, when the studies were broken down into different age categories, some categories were more represented than others, depending on the compartmental volume. A statistical comparison between age categories was thus not possible. We therefore instead present a descriptive overview of the current state of the literature with regard to the representation of the examination of sex differences across different age categories.

Data were split into six categories. The first – *infant* – includes data from newborns to 1 year-olds, the second – *early childhood* – covers 2–6 year-olds, the third – *late childhood* – includes 7–17 year-olds, the fourth – *mature* – is made up of 18–59 year-olds, the fifth – *senior* – included individuals over 60 years old, and lastly a six category – *lifespan* – encompasses studies with wide age ranges (encompassing more than 2 of the above age categories), e.g. spanning from infancy, mid-teens or early twenties up to the seventh or eighth decade of life (e.g. Courchesne et al., 2000; Good et al., 2001a; Hoogendam et al., 2012).

**Fig. 3:**
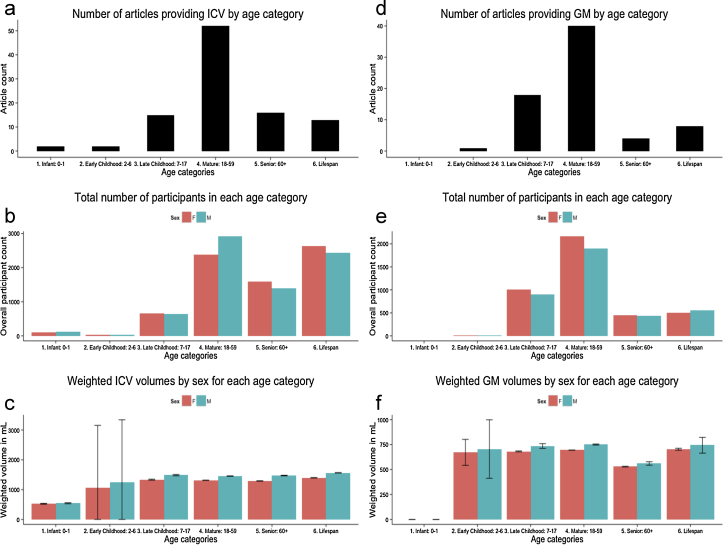
Breakdown by age categories for reports providing intracranial volume and grey matter volume. Three plots display the breakdown of studies examining intracranial volume (ICV) and grey matter volume (GM) in the current literature across six age categories: ‘infant’ (0–1 years), ‘early childhood’ (2–6 years), ‘late childhood’ (7–17 years), ‘mature’ (18–59 years), ‘senior’ (60+ years), and ‘lifespan’ (any study covering more than 2 age ranges): (a) the total number of articles providing ICV in each age category; (b) the sum of the total number of male and female participants included in those age categories; and (c) displays the weighted mean volumes of ICV and weighted error bars for males and females across all age categories. (d) The total number of articles providing GM in each age category; (e) the sum of the total number of male and female participants included in those age categories; and (f) displays the weighted mean volumes of GM and weighted error bars for males and females across all age categories.

Fig. 3 gives a descriptive overview of the articles providing ICV (Fig. 3a–c) and GM (Fig. 3d–f) and Supplementary Figures 14–18 give an overview of TBV, Cb, WM, CSF and Cbl respectively. As can be seen from Fig. 3a,d and Supplementary Figures 14a–18a, the ‘mature’ age category is best represented with by far the largest number of studies across all volumes. In addition, the ‘infant’ and ‘early childhood’ categories are sometimes empty, showing that these age groups and others are underrepresented in this meta-analysis.

Fig. 3b,e and Supplementary Figures 14b–18b display the sum of the total number of male and female participants across all the studies in each age category. From this it is again apparent that the ‘mature’ category is best represented, and depending on the volume, the next best representations are in the ‘late childhood’, ‘senior’ and ‘lifespan’ categories. However, since the number of studies in those categories are still much lower than in the ‘mature’ age category but the number of participants are still quite high, this may suggest larger sample sizes in studies examining sex differences in ‘late childhood’, ‘senior’ and ‘lifespan’ categories.

Lastly, Fig. 3c,e and Supplementary Figures 14c–18c show the weighted volume and weighted error bars for each compartmental volume per sex. From these graphs it is apparent that the size of the error bars significantly depends on the number of studies and subjects in each age category. When taking into account the widely various number of articles and subjects in each age bin it would not be statistically valid to compare volumes across the different age categories. In addition, these graphs indicate that the meta-analytic overall volume results may be skewed towards sex differences present in the 18–45 years old ‘mature’ age-range.

### 3.3 Regional sex differences in volume and tissue density

Table 2 and Supplementary Tables 2 and 3 show that studies included in the meta-analyses are substantially different with respect to sample size, age range, image acquisition parameters, statistical models and thresholds. The GPR-CMBA estimates the extent/variance of such an inconsistency/heterogeneity in our study pool, similar to RFX variance in hierarchical models.

**Fig. 4:**
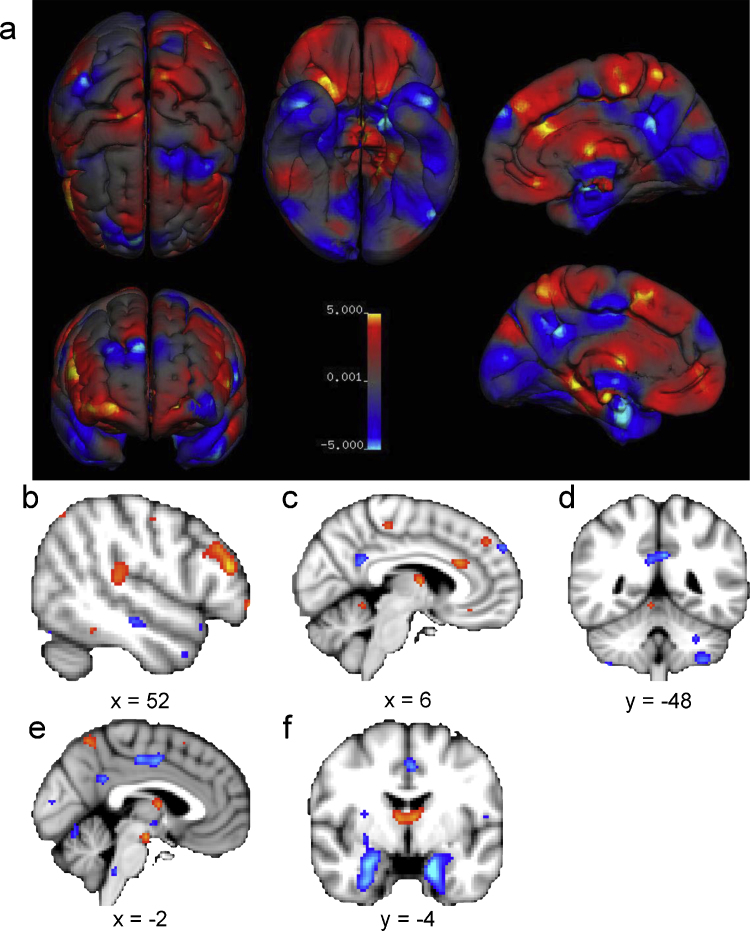
Voxel-based regional sex differences in grey matter volume. Female > Male in red, and Male > Female is in blue. Panel a, rendered overview of uncorrected regional sex differences in grey matter volume. All other panels are thresholded at FDR *q* < 0.01. Panels b–f display areas of larger volume in females (red) including (b) the right inferior and middle frontal gyri, pars triangularis and planum temporale; (c) thalamus and right anterior cingulate gyrus; and (f) left and right thalamus; and areas of larger volume in males (blue), including (c) the anterior cingulate gyrus; (d) bilateral posterior cingulate gyrus and precuneus and left cerebellum; (e) anterior and posterior cingulate gyri; and (f) left and right amygdalae, hippocampi and parahippocampal gyri.

**Fig. 5:**
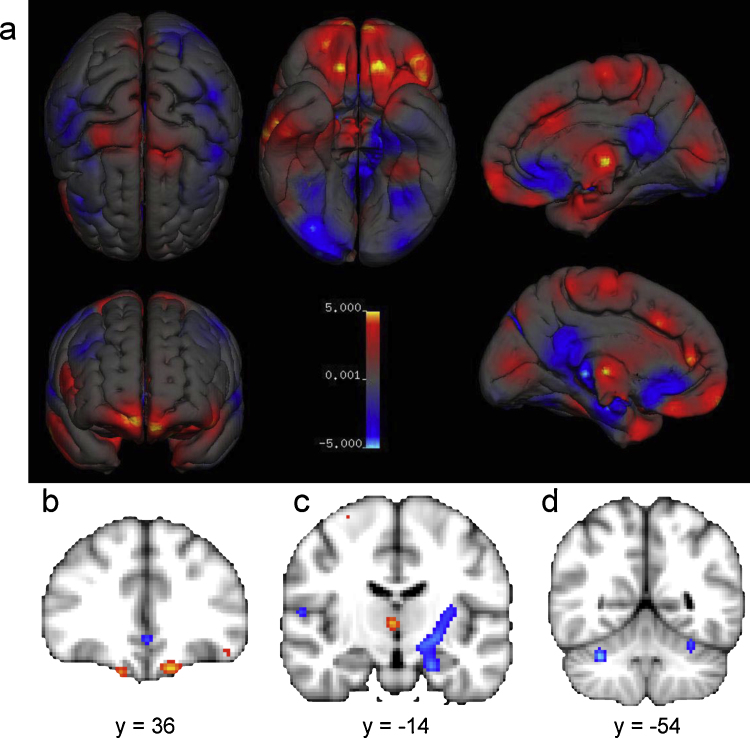
Voxel-based regional sex differences in grey matter density. Female > Male in red, and Male > Female is in blue. Panel a, rendered overview of uncorrected regional sex differences in grey matter concentration. All other panels are thresholded at FDR *q* < 0.05. Panels b–c display areas of larger volume in females (red) in (b) frontal pole and (c) right thalamus; and in males (blue) including (c) left amygdala, hippocampus, insular cortex and putamen; (d) right and left cerebellum VI lobe.

Group difference information for location (*x*, *y*, *z* coordinates in either Montreal Neurological Institute (MNI) or Talairach anatomical spaces) and effect size information (*P*-values, Cohen's *d*, Pearson's *r*, *f*^2^-, *T*-, or *Z*-statistics) were gathered for all reported data points (or foci) of source studies. Reported statistics were converted into *Z*-statistics and coordinates were transformed to MNI space when necessary. Meta-analyses results were all reported in MNI space on a *Z*-map thresholded at their respective FDR-corrected *Z*-value, see Table 4 for results of GM volume and tissue density. For uncorrected meta-analytic summary images and FDR-corrected images of the key results, see Fig. 4 for volume and Fig. 5 for density.

#### 3.3.1 Regional volume meta-analysis

All 16 studies included in the volume voxel-based meta-analysis included a between-group comparison of GM volume, leading to a total of 264 reported GM foci. Only 4 studies performed a WM volume comparison, with a total of 30 WM foci. Since 30 data points are insufficiently spatially dense to perform a meta-analysis, only a coordinate-based meta-analysis on GM volume is currently possible. The 16 studies provided a total of 2186 brains (49% female) aged between 7 and 80 years old. Because an FDR-correction at voxel-level *q* = 0.05 gave diffuse spatial results, we opted for a more stringent correction to capture the most reliable group differences. The (FDR *q* = 0.01) thresholded *Z*-value was 3.428 for the male > female contrast and 3.616 for the female > male contrast, and results are reported in Table 4 using an extent threshold of 60 continuous voxels.

On average, males have larger grey matter volume in bilateral amygdalae, hippocampi, anterior parahippocampal gyri, posterior cingulate gyri, precuneus, putamen and temporal poles, areas in the left posterior and anterior cingulate gyri, and areas in the cerebellum bilateral VIIb, VIIIa and Crus I lobes, left VI and right Crus II lobes. Females on average have larger volume at the right frontal pole, inferior and middle frontal gyri, pars triangularis, planum temporale/parietal operculum, anterior cingulate gyrus, insular cortex, and Heschl's gyrus; bilateral thalami and precuneus; the left parahippocampal gyrus and lateral occipital cortex (superior division).

#### 3.3.2 Regional tissue density meta-analysis

Eight of the nine studies (eight of the ten age-matched groups) investigating voxel-based sex differences in brain tissue density performed a GM analysis, with a total of 86 reported foci. Only three performed a WM density analysis with a total of 13 foci again discouraging a meta-analysis. The eight studies provided a total number of 741 brains (53% female), aged between 10 and 81 years. Results are reported (with FDR *q* = 0.05). *Z*-values were 3.247 for the male > female contrast and 3.445 for the female > male contrast, reported in Table 4 with an extent threshold of 60 continuous voxels. Areas of higher GM density in males compared to females included the left amygdala, hippocampus, insular cortex, pallidum, putamen, claustrum, and an area in the right VI lobe of the cerebellum. The left frontal pole has significantly higher GM tissue density in females compared to males.

## 4 Discussion

This meta-analysis collated and quantified current literature regarding sex differences in human brain morphology. Our first aim was to examine in what way and where typically developing male and female brains differ. Furthermore we explored the question that if male and female brains differ, do such areas of differences overlap with areas commonly implicated in psychiatric conditions? We found that across a wide age range, from newborns to individuals over 80 years old, differences in overall brain volumes are sustained between males and females. On average males have larger ICV (12%), TBV (11%), Cb (10%), GM (9%), WM (13%), CSF (11.5%) and Cbl (9%) absolute volumes than females. In addition, the ‘mature’ (18–59 years old) age category is best represented with by far the largest number of studies across all volumes and may thus have skewed the meta-analytic results.

At a regional level, males on average have larger volumes and higher tissue densities in the left amygdala, hippocampus, insular cortex, putamen; higher densities in the right VI lobe of the cerebellum and in the left claustrum; and larger volumes in the bilateral anterior parahippocampal gyri, posterior cingulate gyri, precuneus, temporal poles, and cerebellum, areas in the left posterior and anterior cingulate gyri, and in right amygdala, hippocampus, and putamen. Females have on average higher density in the left frontal pole, and larger volumes in the right frontal pole, inferior and middle frontal gyri, pars triangularis, planum temporale/parietal operculum, anterior cingulate gyrus, insular cortex, and Heschl's gyrus; bilateral thalami and precuneus; the left parahippocampal gyrus and lateral occipital cortex (superior division).

The results from the regional volume and density analyses mostly include areas that are part of the limbic and language systems. Additionally, they also indicate a potential lateral asymmetry in sex differences. Volume increases in males are mostly in bilateral limbic areas and left posterior cingulate gyrus, whereas higher densities are mostly limited to the left side of the limbic system. On the other hand, larger volumes in females were most pronounced in areas in the right hemisphere related to language in addition to several limbic structures such as the right insular cortex and anterior cingulate gyrus. Despite this seeming sex difference in patterns of lateralization, it was unfortunately not possible to statistically, directly examine sex differences in asymmetry in this meta-analysis due to the limited number of articles performing a voxel-wise asymmetry analysis. Existing literature employing region-of-interest analyses (Chiarello et al., 2009; Sommer et al., 2008) have provided further exploration to this issue. Given the rich evolutionary and neurobiological implications in sex differences and brain lateralization, future studies on sex differences in human neuroanatomy should investigate patterns of asymmetry in a whole-brain framework (Crow et al., 2013; Good et al., 2001b; Fan et al., 2010).

### 4.1 Brain development

Recent studies have shown different developmental trajectories for regional volumes as well as for compartmental volumes (Brain Development Cooperative Group, 2012; Good et al., 2001b; Koolschijn and Crone, 2013; Lenroot et al., 2007; Li et al., 2014; Pfefferbaum et al., 2013;). Longitudinal studies on specific neuroanatomical structures usually show sex and age effects, but not necessarily sex by age interaction effects, on trajectories for most of the structures we found to be different between males and females in this meta-analyses (e.g., the amygdala, hippocampus, putamen, precuneus, and thalamus) in adulthood (Li et al., 2014) and during adolescence (Brain Development Cooperative Group, 2012; Koolschijn and Crone, 2013; Lenroot et al., 2007).

We recognize the limitations of the existing literature in our study in providing a descriptive account across six age categories for overall volumes. We were not able to perform statistical tests comparing volume differences between age groups due to heterogeneous sample sizes: Fig. 3 and Supplementary Figures 14–18 show a bias in the number of studies examining sex differences in the 18–45 year-old ‘mature’ age categories. Future research should explore sex differences in other age categories separately, and more importantly, across time using longitudinal designs to provide a better understanding of the development of total brain volumes across the lifespan.

### 4.2 Potential implications for understanding neuropsychiatric conditions

The findings in this study may serve as a foundation for future studies by providing sex-differential norms of brain volume and density information. Studying sex differences in regional and overall brain volumes could also provide clues about how biological, environmental and gene-environment interaction mechanisms associated with sexual differentiation shape brain development. Previous studies found significant correlations of hormones on regional and overall sex differences in brain volume in children (Lombardo et al., 2012), adolescents (Herting et al., 2012; Paus et al., 2010; Witte et al., 2010) and adults (Lentini et al., 2013; Pletzer et al., 2010). Genetic influences, such as variation in the number of CAG repeats in the androgen receptor gene (Raznahan et al., 2010) and sex-biased gene expression (Hawrylycz et al., 2012; Kang et al., 2011), also have an impact on (cortical) brain development. In addition, environmental influences such as birth weight (Raznahan et al., 2012) and effects of prenatal nutrition, which can influence DNA methylation of insulin-like growth factors (Heijmans et al., 2008), affect general (brain) development as well (Hansen-Pupp et al., 2011).

The majority of the regions displaying sex differences in this meta-analysis also show structural differences between typically developing individuals and individuals with neuropsychiatric conditions (areas of the limbic system, e.g., amygdala, hippocampus and insula) such as autism (Beacher et al., 2012; Cauda et al., 2011; Lai et al., 2013), depression (Bora et al., 2012), schizophrenia (Shepherd et al., 2012) and attention deficit hyperactivity disorder (Etkin and Wager, 2007), providing some bases for the hypothetical view that factors driving the development of typical sex differences might also play a role in the emergence of these neuropsychiatric conditions. Most of these conditions are neurodevelopmental and their prevalence may change over developmental periods. For example, autism has a male bias from childhood onwards, higher prevalence and earlier age of onset for schizophrenia are reported for males, whereas for depression and anxiety disorder the prevalence doubles in girls during adolescence (Rutter et al., 2003). From these we could speculate that sexually differentiating mechanisms may be involved in the neurodevelopment of individuals who develop these psychiatric conditions. Therefore, research investigating differences in brain structure in psychiatric conditions that are asymmetrically affected by sex should stratify samples by sex and perform within sex case–control comparisons.

On a different note, stratifying by sex may also be important for studies measuring for regional cerebral blood flow (rCBF), such as in positron emission topography (PET) studies, since the size distribution volume of the area of interest may differ for males and females. Although sex differences in brain function have previously been reported and reviewed (Sacher et al., 2013; Stevens and Hamann, 2012), the link between function and structure is still under-explored; no predictions as to how structure may influence physiology or behaviour are possible from these meta-analyses.

### 4.3 Limitations

Several limitations regarding the sample size of the meta-analyses and individual study parameters should be acknowledged. First, the total volume analyses were all performed on absolute brain volumes. Most studies report absolute volumes rather than values adjusted for weight and/or height. The overall volume analyses are thus a reflection of the existing literature. As it could still be debated what the implications are by brain size ‘adjusted’ for body weight and/or height, and whether body weight and/or height (males are on average taller than females) influence brain size, the present results should be interpreted in light of the conventional ways of report in the literature. Future studies also investigate brain volumes adjusted for weight and height in addition to absolute volumes.

Second, we recognize that no definite statistical inference can be made from the analyses by age categories due to (1) too small sample sizes to perform volume meta-analyses in each age category and (2) heterogeneous age-range of the categories (e.g. the ‘mature’ category spans over 42 years). This reflects the limitation of the current literature. Longitudinal follow-up design is the only way to adequately address lifespan brain development, including how sex differences play a role.

Third, in the extant literature it is not always made clear if individual studies include the cerebellum and/or brainstem in their analysis. Although significant sex differences were found in cerebellum volume and thus sex differences may still be present, this inconsistency in the literature could affect results on total white or grey matter volumes.

Lastly, even though GPR-CMBA takes into account heterogeneity between studies, the variation in smoothing, sample size, and covariates in statistical models can all influence voxel-based morphometry analyses (Barnes et al., 2010; Shen and Sterr, 2013) and act as important sources of statistical noise.

### 4.4 Conclusion and future research

In summary, this study provides the first meta-analysis of sex differences in overall and regional brain volumes and regional brain tissue densities. Future research should test whether sex differences in brain structure underlies skewed sex ratios of neurological and psychiatric conditions and whether brain areas affected in such conditions are caused by physiological mechanisms associated with the development of typical sex differences. For example, recent studies show that sex differences in the adult (Hawrylycz et al., 2012) and developing (Kang et al., 2011) brain transcriptome could be analyzed in conjunction with neuroanatomy, to examine if sexually differentiated brain structures are driven by differences in the brain transcriptome, sex chromosomal and/or environmental effects.

## Figures and Tables

**Fig. 2:**
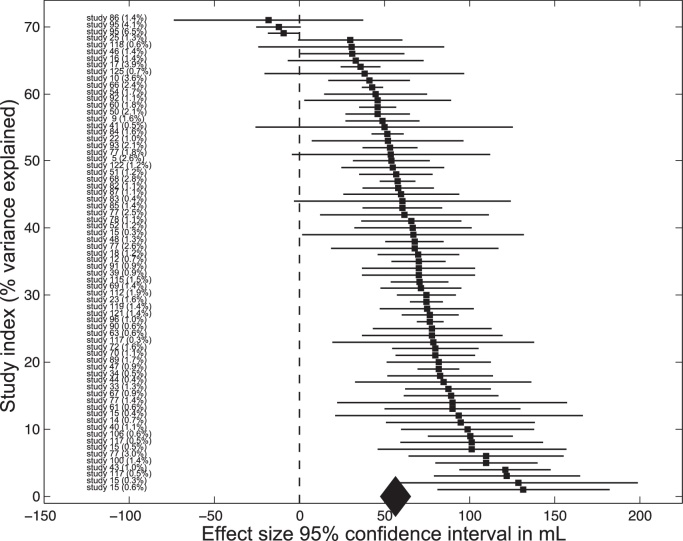
Forest plot for the grey matter volume meta-analysis. Overview of all studies included in the grey matter volume meta-analysis. The square indicates the effect size in mL of each study (i.e. the difference in mL volume between males and females) and the bars indicate the 95% confidence interval of each study. The studies corresponding to the effect size can be found on the left. Study IDs correspond to the study IDs in Table 1. The diamond at the bottom of the figure indicates the meta-analytic effect size and its variance.

**Table 1:** Studies included in the overall volumes meta-analyses.

ID	Study	Year	*N* (F)	Age			Volumes
				Mean (SD)	Range	Category	
1	Abbs	2011	48 (21)	40.5^a^ (10.8^a^)	–	4	Cb
2	Acer	2008	53 (27)	–	20–25	4	Cbl
3	Allen	1991	24 (12)	9.1^a^ (3.8^a^)	2–15	6	Cb
			122 (61)	42^a^ (13.8^a^)	16–79	6	
4	Allen	2002	46 (23)	32.4^a^ (8.2^a^)	22–49	4	Cbl
5	Allen	2003	46 (23)	32.4^a^ (8.2^a^)	22–49	4	Cb, GM, WM
6	Andreasen	1994	90 (42)	27.4 (10.3)	–	4	TBV, CSF, Cbl
7	Arango	2008	66 (32)	34.1^a^ (7.3^a^)	23–51	4	ICV, TBV, CSF
8	Baibakov	2010	60 (30)	1^a^ (0.08^a^)	0–1	1	TBV
9	Barnes	2010	82 (34)	–	33–35	4	GM
10	Barrick	2005	30 (15)	28.8^a^	–	4	Cb, GM, WM
11	Barta	2003	30 (15)	39.1^a^ (10.5^a^)	–	4	TBV
12	BDCG	2012	325 (173)	10.9^a^ (3.8^a^)	4–18	3	TBV, GM, WM, Cbl
13	Beacher	2012	30 (15)	30^a^ (8^a^)	–	4	WM
14	Blanton	2004	46 (25)	10.9^a^ (2.9^a^)	6–17	3	ICV, GM, WM
15	Blatter	1995	44 (20)	22.3^a^ (2.2^a^)	16–25	4	ICV, TBV, GM
			43 (24)	30.8^a^ (3.2^a^)	26–35	4	WM, CSF
			38 (22)	40.9^a^ (2.9^a^)	36–45	4	
			39 (24)	50.6^a^ (2.7^a^)	46–55	4	
			30 (15)	60.2^a^ (2.6^a^)	56–65	4	
16	Bloss	2007	27 (14)	3.7^a^ (1.1^a^)	1–5	2	ICV, TBV, GM, WM, Cbl
17	Boes	2008	117 (56)	12.3^a^ (2.8^a^)	7–17	3	GM
18	de Bruin	2005	91 (44)	49.7^a^ (7.9^a^)	–	4	ICV, Cb, GM, WM, CSF, Cbl
19	Bryant	1999	37 (18)	33.8^a^ (7.3^a^)	21–45	4	ICV
20	Buckner	2004	168 (88)	29.1^a^ (11.7^a^)	–	4	ICV
			77 (57)	76.8^a^ (8.8^a^)	–	5	
21	Carne	2006	97 (49)	33.7 (13.6)	15–69	6	TBV, Cb, Cbl
22	Caviness	1996	30 (15)	9.2^a^	7–11	3	ICV, Cb, GM, WM, Cbl
23	Chen	2007	411 (227)	46.7^a^ (1.4^a^)	44–48	4	ICV, GM, WM, CSF
24	Chen	2012	27 (15)	22.6^a^ (1.8^a^)	–	4	TBV
25	Cheng	2009	50 (25)	27.1^a^ (9.4^a^)	19–50	4	TBV, GM, WM
26	Choi	2010	43 (21)	24.5^a^ (4^a^)	–	4	ICV
27	Chung	2005	118 (60)	23 (2.6)	–	4	Cbl
			100 (59)	47.5 (3.7)	–	4	
28	Clayden	2011	59 (34)	11.5 (2.1)	8–16	3	ICV, WM
29	Coffey	1998	330 (201)	75 (5.1)	66–96	5	Cb
30	Collinson	2003	30 (12)	16.4 (1.7)	–	3	Cb
31	Courchesne	2000	116 (37)	21.4 (20)	1–80	6	ICV
32	Cowell	2007	S, 18 (8)	35.3^a^ (3.3^a^)	20–49	4	ICV
			S, 16 (10)	57.8^a^ (2.5^a^)	50–72	5	
			L, 19 (8)	35.3^a^ (3.1^a^)	20–49	4	
			L, 15 (10)	58.5^a^ (2.5^a^)	50–72	5	
33	Crespo-Facorro	2011	76 (31)	28.1^a^ (7.6^a^)	15–51	4	ICV, GM
34	De Bellis	2001	118 (57)	11.9^a^ (2.4^a^)	6–17	3	ICV, Cb, GM, WM
35	DeCarli	2005	1181 (233)	–	34–54	4	ICV
			517 (288)	–	55–61	4	
			546 (275)	–	62–70	5	
			487 (272)	–	71–96	5	
36	Duggal	2005	30 (15)	30^a^ (9.5^a^)	–	4	ICV
37	Edland	2002	184 (121)	79^a^ (6.5^a^)	–	5	ICV
38	Eliez	2001	85 (64)	10.6^a^ (2.9^a^)	–	3	Cb, GM, WM, CSF
40	Fahim	2012	38 (20)	8.4^a^ (0.1^a^)	–	3	GM, WM, CSF
41	Filipek	1994	20 (10)	27.2^a^ (5.2^a^)	17–37	4	TBV, Cb, GM, WM, Cbl
42	Frederikse	1999	30 (15)	39.1^a^	23–58	4	TBV
43	Garcia-Falgueras	2006	91 (51)	20.1^a^ (2.8^a^)	18–33	4	ICV, GM, WM, CSF
44	Ge	2002	54 (22)	46.8 (19.3)	20–86	6	ICV, GM, WM
45	Gilmore	2007	74 (34)	0.8^a^ (0.03^a^)	0.7–0.9	1	ICV, Cbl
46	Goldstein	2001	48 (21)	39.8 (11.4^a^)	–	4	Cb, GM, WM
47	Good	2001a	465 (200)	32^a^ (12.2^a^)	17–79	6	GM, WM, CSF
48	Guo	2008	158 (80)	15 (4.7)	7–22	6	ICV, GM, WM
49	Gur	1991	69 (35)	42.9^a^ (20.4^a^)	18–80	6	TBV, CSF
50	Gur	1999	80 (40)	26^a^ (5.5^a^)	18–45	4	ICV, TBV, GM, WM, CSF
51	Gur	2002	116 (59)	26^a^ (5.5^a^)	18–49	4	ICV, GM, WM, CSF
52	Hänggi	2010	43 (25)	22.3 (2.5)	19–32	4	ICV, GM, WM, CSF
53	Hogan	2011	228 (107)	68.7^a^ (0.7^a^)	68–69	5	ICV
54	Hommer	2001	39 (19)	40.8^a^ (7.7^a^)	–	6	ICV, GM, WM
55	Hoogendam	2012	3962 (2156)	60.1 (8.5)	>45	6	ICV
56	Hutchinson	2003	120 (60)	25.1^a^ (4.6^a^)	–	4	TBV, Cbl
57	Jenkins	2000	52 (28)	56.1 (12.1)	36–85	6	ICV
58	Knaus	2004	24 (12)	31.4 (8.9)	21–53	4	ICV
59	Koscik	2009	76 (38)	26.7^a^ (7.2^a^)	18–47	4	Cb
60	Kruggel	2006	290 (145)	24.2^a^ (2.8^a^)	18–32	4	ICV, TBV, GM, WM, CSF
61	Lai	–	60 (30)	27.9^a^ (6.1^a^)	18–49	4	ICV, TBV, GM, WM, CSF
62	Lavretsky	2004	41 (20)	72.2 (7.3)	–	5	ICV
63	Lee	2004	62 (28)	–	18–77	6	ICV, GM, WM
64	Lee	2009a	57 (29)	24.4^a^ (2.1^a^)	20–28	4	ICV, Cbl
			58 (26)	67.8^a^ (3.1^a^)	62–74	5	
65	Lee	2009b	68 (39)	21.7 (2.3)	20–29	4	ICV
66	Lemaître	2005	662 (331)	69.5^a^ (3^a^)	63–75	5	ICV, GM, WM, CSF
67	Lentini	2012	86 (45)	35.1^a^ (7.1^a^)	26–51	4	ICV, TBV, GM, WM, CSF
68	Leonard	2008	200 (100)	21.6^a^	–	4	ICV, GM, WM, CSF, Cbl
69	Li	2012	76 (38)	43^a^ (15.9^a^)	19–70	6	TBV, GM
70	Luders	2002	100 (50)	24.7^a^ (4.4^a^)	–	4	ICV, GM, WM, CSF
71	Luders	2003	59 (29)	24.3^a^ (5^a^)	–	4	ICV
72	Luders	2005	60 (30)	24.8^a^ (4.5^a^)	–	4	ICV, GM, WM, CSF
73	Maller	2006	150 (74)	62.3^a^ (1.4^a^)	60–64	5	TBV
74	Mann	2011	70 (35)	54.5^a^ (20.3^a^)	20–87	6	Cb
75	Matsumae	1996	12 (6)	–	24–40	4	ICV, TBV,
			15 (5)	–	41–60	4	CSF
			22 (12)	–	61–80	5	
76	Mitchell	2003	100 (56)	32.6 (12.3)	14–68	6	Cb
77	Mortamet	2005	10 (5)	–	20–29	4	ICV, GM,
			10 (5)	–	30–39	4	WM, CSF
			10 (5)	–	40–49	4	
			10 (5)	–	50–59	4	
			10 (5)	–	60–69	5	
78	Narr	2007	65 (35)	28.2^a^ (7.3^a^)	–	4	ICV, GM, WM, CSF
79	Neufang	2009	46 (23)	11.3^a^ (2.2^a^)	8–15	3	TBV
80	Nopoulos	1997	80 (40)	28.1^a^ (7.4^a^)	–	4	TBV, CSF
81	Nopoulos	2000	84 (42)	23.3^a^ (3.3^a^)	19–31	4	ICV, Cb, CSF, Cbl
82	Nunnemann	2009	133 (73)	–	29–80	6	ICV, GM
83	Passe	1997	43 (30)	53.7^a^ (17.9^a^)	24–82	6	TBV, GM, WM
84	Paus	2010	419 (215)	15.3^a^ (1.92^a^)	–	3	ICV, GM, WM
85	Peper	2009	78 (41)	11.9^a^ (1.1^a^)	10–14	3	ICV, GM, WM, Cbl
86	Hulshoff Pol	2006	15 (6)	24.2^a^ (7.3^a^)	16–50	4	ICV, TBV, GM, WM
87	Ponseti	2007	49 (25)	25.1^a^ (3.3^a^)	–	4	GM, WM, CSF
88	Qiu	2013	161 (74)	0.03^a^ (0.02^a^)	–	1	ICV
89	Rametti	2011	38 (19)	32.5^a^ (7.2^a^)	–	4	ICV, GM, WM, CSF
90	Reig	2011	94 (33)	15.4 (1.4)	9–19	3	ICV, GM, WM, CSF
91	Reiss	1996	85 (64)	10.6^a^ (2.9^a^)	5–17	3	ICV, GM, WM, CSF
92	Reiss	2004	31 (17)	8.5 (0.7)	7–11	3	GM, WM, CSF
93	Resnick	2000	116 (48)	70.4 (7.5)	59–85	5	TBV, GM, WM
94	Rhyu	1999	20 (11)	–	20–29	4	Cbl
			19 (9)	–	30–39	4	
			23 (13)	–	40–49	4	
			20 (10)	–	50–59	4	
			23 (12)	–	60–69	5	
			19 (12)	–	70–79	5	
95	Riello	2005	133 (95)	–	≤60	4	ICV, GM,
			96 (63)	–	>60	5	WM
96	Rijpkema	2012	1004 (591)	22.8^a^ (3.4^a^)	18–36	4	GM, WM
97	Sachdev	2008	383 (172)	62.7^a^ (1.4^a^)	60–64	5	ICV
98	Salinas	2012	108 (54)	12.3^a^ (2.9^a^)	7–17	3	Cb
99	Sandu	2008	18 (10)	13.8 (0.5)	–	3	ICV
100	Savic	2011	48 (24)	34^a^ (6^a^)	26–48	4	ICV, TBV, GM, WM
101	Schlaepfer	1995	60 (17)	31.6^a^ (7.9^a^)	–	4	ICV
102	Sgouros	1999	33 (15)	2.1^a^ (1.6^a^)	0–4	2	ICV
			11 (3)	8.1^a^ (1.5^a^)	5–9	3	ICV
			24 (8)	12.9^a^ (1.5^a^)	10–15	3	ICV
103	Shan	2005	13 (8)	27.4^a^ (4.9^a^)	–	4	TBV
			13 (8)	69.8^a^ (1.9^a^)	–	5	
104	Shin	2005	30 (15)	27.2^a^ (6.3^a^)	–	4	ICV
105	Soloff	2008	30 (19)	25.6 (7.7)	–	4	ICV
106	Sowell	2007	176 (86)	32.4^a^ (21.8^a^)	7–87	6	ICV, GM, WM, CSF
107	Sparks	2002	26 (8)	4 (0.5)	3–4	2	Cb, Cbl
108	Sullivan	2001	92 (41)	46.9^a^ (15.1^a^)	22–71	6	ICV
109	Sullivan	2004	143 (48)	49.3^a^ (16.9^a^)	20–85	6	ICV
110	Sullivan	2005	128 (44)	–	20–85	6	ICV
111	Takahashi	2004	61 (31)	24.5 (5.5)	18–38	4	ICV
112	Takao	2010	109 (58)	26.3^a^ (2.1^a^)	21–29	4	GM, WM
113	Tepest	2010	29 (11)	33 (9.1)	20–55	4	TBV
114	Uematsu	2012	27 (9)	–	0–2	1	TBV
			34 (17)	–	2–10	2	
			48 (26)	–	10–25	6	
115	Wang	2012	140 (70)	20.9^a^ (1.8^a^)	18–26	4	ICV, GM, WM, CSF
116	Whitwell	2001	55 (31)	–	23–83	6	ICV
117	Wilke	2007	67 (34)	7.6^a^ (1.2^a^)	–	3	GM, WM
			66 (34)	11.1^a^ (1^a^)	–	3	
			67 (34)	15.6^a^ (1.7^a^)	–	3	
118	Witte	2010	34 (17)	26.6 (5)	21–47	4	ICV, GM, WM, CSF
119	Wood	2008a	60 (30)	28.9^a^ (8.4^a^)	18–50	4	ICV, GM
120	Wood	2008b	74 (37)	12.5^a^ (2.9^a^)	7–17	3	ICV, Cb
121	Yamasue	2008	155 (66)	28.5^a^ (4.2^a^)	21–40	4	ICV, GM, WM, CSF
122	Yang	2008	57 (23)	11.7	7–17	3	ICV, GM, WM, CSF
123	Yoshii	1988	58 (29)	56.3 (17.5)	21–81	6	ICV
124	Ystad	2009	O, 84 (60)	65.1	47–75	5	ICV, Cb
			B, 86 (60)	59.3	46–77	5	
125	Yurgelun-Todd	2002	30 (20)	14.7 (1.5)	13–17	3	ICV, GM, WM
126	Zhang	2007	24 (12)	27.7 (4.4)	17–35	4	WM
			12 (7)	74.8 (2.6)	72–80	5	

*Abbreviations*: B, Bergen; BDCG, brain development cooperative group; Cb, cerebrum (excluding CSF and Cbl); Cbl, cerebellum; CSF, cerebrospinal fluid; F, female; GM, grey matter; ICV, intracranial volume (including CSF); ID, identification number; L, Liverpool; N, number of participants; O, Oslo; S, Sheffield; SD, standard deviation; TBV, total brain volume (including Cbl but excluding CSF); WM, white matter.

a Weighted mean and standard deviations.

**Table 2:** Studies included in the regional meta-analyses.

ID	Study	Year	*N* (F)	Age			Volume	No. peak voxels
				Mean (SD)	Range	Category		
*Regional volume meta-analysis*								
127	Almeida	2009	55 (32)	±31	–	4	GM	9
23	Chen	2007	411 (227)	46.7^a^ (1.4^a^)	44–48	4	GM	11
25	Cheng	2009	50 (25)	27.1^a^ (9.4^a^)	19–50	4	GM	14
128	Fan	2010	112 (46)	24.7^a^ (2.8^a^)	18–33	4	Cbl GM	14
47	Good	2001a	465 (200)	32^a^ (12.2^a^)	17–79	6	GM; WM	18; 7
48	Guo	2008	158 (80)	15.3 (4.7)	7–22	6	GM	14
61	Lai	–	60 (30)	27.9^a^ (6.1^a^)	18–49	4	GM; WM	42; 18
67	Lentini	2012	86 (45)	35.1^a^ (7.1^a^)	26–51	4	GM; WM	8; 2
129	Lombardo	2012	217 (116)	9.5^a^ (1.1^a^)	8–11	3	GM	31
82	Nunnemann	2009	133 (73)	–	29–80	6	GM	32
130	Pletzer	2010	28 (14)	25.6^a^ (4.7^a^)	–	4	GM	25
100	Savic	2011	48 (24)	34^a^ (6^a^)	26–48	4	GM; WM	7; 3
131	Welborn	2009	117 (58)	22.8	18–40	4	GM & WM	7
118	Witte	2010	34 (17)	26.6 (5)	21–47	4	GM	18
121	Yamasue	2008	155 (66)	28.5^a^ (4.2^a^)	21–40	4	GM	12
122	Yang	2008	57 (23)	11.7	7–17	3	GM	2

*Regional density meta-analysis*								
132	Dong	2010	40 (20)	–	20–25	4	GM; WM	3; 3
43	Garcia-Falgueras	2006	91 (51)	20.1^a^ (2.8^a^)	18–33	4	GM	14
47	Good	2001a	465 (200)	32^a^ (12.2^a^)	17–79	6	WM	9
40	Fahim	2012	38 (20)	8.4^a^ (0.1^a^)	–	3	GM; WM	3; 1
133	Lord	2010	46 (31)	–	50–74	5	GM	11
85	Peper	2009	78 (41)	11.9^a^ (1.1^a^)	10–15	3	GM	17
134	Takahashi	2011	91 (35)	31.4^a^ (8^a^)	20–79	6	GM	15
			136 (81)	65.7^a^ (8.4^a^)		5		4
135	Van Laere	2001	81 (41)	44.2	20–81	6	GM	3
115	Wang	2012	140 (70)	20.9^a^ (1.8^a^)	18–26	4	GM	16

*Notes*: Study ID count corresponds to and continues on from Table 1. *Abbreviations*: F, female; GM, Grey matter; ID, identification number; *N*, number of participants; No., number; SD, standard deviation; WM, white matter.

a Weighted mean and standard deviations.

**Table 3:** Results of the overall volumes meta-analyses.

Volume	aStudies	bGroups	*N* (F^c^)	Analysis type	*Q*	*I*^2^	Outcome	Cohen's d	Percentage difference	dMean difference	CI 95%	*p*-Value
ICV	77	100	14,957 (48%)	FFX	62.3	0	M > F	3.03	12%	135.3	117.8–152.8	<10^−6^
TBV	31	40	2532 (50%)	RFX	92.8	57.9	M > F	2.1	10.8%	131	92.1–170.0	<10^−6^
Cb	22	24	1851 (54%)	FFX	12.5	0	M > F	3.35	9.8%	51.06	38.7–63.5	<10^−6^
GM	60	71	7934 (52%)	FFX	26.5	0	M > F	2.13	9.4%	56.51	44.2–68.9	<10^−6^
WM	57	69	7515 (52%)	FFX	39.9	0	M > F	2.06	12.9%	44.4	34.2–54.6	<10^−6^
CSF	35	45	4484 (50%)	FFX	15.5	0	M > F	1.21	11.5%	18.72	9.6–27.8	3.2 × 10^−5^*
Cbl	19	26	1842 (51%)	FFX	9.7	0	M > F	1.68	8.6%	7.78	4.2–11.4	1.4 × 10^−5^*

*Abbreviations*: Cb, cerebrum; Cbl, cerebellum; CSF, cerebrospinal fluid; F, females; FFX, fixed effects model; GM, grey matter; *I*^2^, *I*^2^ index; ICV, intracranial volume; *N*, number of subjects; *Q*, Cochran's *Q* test; RFX, random effects model; TBV, total brain volume; WM, white matter.

a Number of studies reporting the respective volumes.

b Number of age-matched gender groups in the analysis (some studies provide more than one sample).

c Number of females in percentages.

d Values reported in mL.

**Table 4:** Sex differences in GM volume and density.

Region	Size^a^	*Z* statistic^b^	*x*	*y*	*z*
**GM volume**					
*Males* *>* *Females*					
Right amygdala, anterior parahippocampal gyrus, hippocampus, putamen and temporal pole	824	7.29	28	−2	−34
		6.89	26	−6	−20
		5.71	26	10	−6
		5.2	26	−14	−26
		4.83	22	6	−12
Left amygdala, anterior parahippocampal gyrus, hippocampus, putamen, temporal pole and orbitofrontal cortex	611	8.76	−16	−4	−26
		5.17	−36	4	−18
		5.67	−28	−12	−18
		5.07	−20	10	−16
Left and right posterior cingulate gyrus and precuneus	225	6.26	−4	−48	30
		5.35	8	−50	24
		4.94	12	−56	32
Left cerebellum, VI, VIIb, VIIIa and Crus I	210	7.82	−28	−60	−32
		4.45	−28	−48	−36
		4.3	−20	−66	−46
Left anterior and posterior cingulate gyrus and precentral gyrus	117	5.93	−2	−6	44
		5.74	−2	−18	46
Left temporal pole	123	5.68	−46	10	−42
		4.69	−50	16	−30
		3.91	−44	24	−30
Right cerebellum, VIIb, VIIIa and Crus I and II	100	5.12	40	−52	−56
		4.37	32	−64	−42
		4.14	28	−56	−46
Left cerebellum VIIIa and VIIb	71	5.17	−34	−48	−54
Right frontal pole	66	5.33	10	60	34
Left temporal pole	60	5.29	−48	−70	−20

*Males* *<* *Females*					
Right inferior frontal gyrus, pars triangularis and pars opercularis, middle frontal gryus, frontal pole	238	7.28	54	34	18
		5.79	46	36	18
		5.34	52	28	28
		4.59	56	20	22
Left and right thalami	182	6.01	−10	−8	16
		5.63	−4	−4	10
		5.41	6	−4	8
Right parietal operculum cortex and planum temporale	115	6.66	54	−28	18
		5.89	54	−28	18
Right anterior cingulate gyrus	85	5.85	8	26	22
Left and right precuneus	79	5.14	−2	−60	60
		4.87	4	−52	64
Right frontal orbital cortex	67	5.07	22	24	−20
		5	20	28	−24
		4.9	28	26	−20
Left posterior parahippocampal gyrus	66	6.92	−14	−36	−6
Left lateral occipital cortex, superior division	64	5.92	−38	−78	32
Right insular cortex and Heschl's gyrus	63	5.78	32	−22	6
		5.27	38	−22	8

**GM density**					
*Males* *>* *Females*					
Left amygdala, hippocampus, insula, pallidum, putamen, claustrum	475	6.42	−20	−12	−22
		6.26	−36	−6	12
		5.78	−26	−10	2
		5.29	−22	−14	−6
		4.56	−30	−18	12
		4.08	−34	−6	−24
Right cerebellum VI	81	6.86	32	−52	−30

*Males* *<* *Females*					
Left frontal pole	64	7.28	−14	38	−24

*Abbreviations*: GM, grey matter; MNI, Montreal Neurological Institute.

a Size in 2 mm voxels on MNI152_T1_2mmbrain mask.

b Values were thresholded at qFDR < 0.01 for GM volume, which is equal to *Z* > *3.44* for M > F and *Z* > *3.62* for F > M, and at qFDR < 0.05 for GM density, which is equal to *Z* > *3.25* for M > F and *Z* > *3.44* for F > M. With a minimum cluster size of 60 voxels. The MNI coordinates indicate the value of the peak voxel and local minima of the cluster and the size the span of the cluster.
