# Supplementary material for: A meta-analysis of sex differences in human brain structure
Source: Neurosci Biobehav Rev. 2014 Feb;39(100):34–50. doi: 10.1016/j.neubiorev.2013.12.004 (PMC3969295; doi:10.1016/j.neubiorev.2013.12.004)
Supplement: Supplementary file 1 [file mmc1.doc]

**Supplementary Data**

**1. Introduction**

In this Supplementary Information we provide supplementary information on the methods and additional data. The methods section will discuss visualization bias in the funnel plots. Lastly, we will present supplementary Tables and Figures.

**2. Supplementary Methods**

*2.1 Biased funnel plots*

As previously mentioned, funnel plots are provided to show heterogeneity and bias in our selected sample of studies for the meta-analysis. In funnel plots (see Supplementary Figures 7-13) the study’s individual effect size (in male-female mL volume difference) is plotted against a measure of study precision, in this case standard error. However, since individual studies differ in their sample size, age range and other factors, this can lead to large differences in their standard error. A symmetrical inverted funnel plot implies a 'well-behaving' dataset, and an asymmetric funnel plot indicates a relationship between effect-size and study size, which may be due publication bias or small-study effects. Asymmetric funnel plots do therefore not necessarily indicate proof of publication bias (Sterne and Egger, 2001).

For example, in Supplementary Figure 8, the funnel plot representing data in the intracranial volume meta-analysis appears to be asymmetrical, with a large portion of the data in one corner of the funnel, and a few studies scattered at the bottom. The two studies that appear to be the largest “outliers” in this plot have study ID’s 116 and 26. They are not actually outliers but the study sample and thus standard error may differ from the other studies considerably.

Study ID 26 corresponds to Choi and colleagues (2010) and study ID 116 belongs to Whitwell and colleagues (2010). Inspecting the data from those studies, it appears that in each study either the male (Choi et al., 2010) or the female (Whitwell et al., 2001) group has a very large standard deviation of the mean ICV volumes (between 810-970 mL). These studies also have large weighted standard deviations, which can be seen in the ICV forest plot as well (see Supplementary Figure 1).

When we reran analysis for the funnel plot again without studies with large standard errors (Choi et al., 2010; Whitwell et al, 2001), we found that the data conforms to a more symmetrical funnel plot without the two studies (see Supplementary Figure 19), showing that individual variability in study samples has a large effect on funnel plot symmetry. Because the data of these two studies does provide information about the population sample and are not real outliers, we did include them in the original intracranial volume meta-analysis.

**3. Supplementary References**

Sterne, J. A. and Egger, M., 2001. Funnel plots for detecting bias in meta-analysis: guidelines on choice of axis. J Clin Epidemiol 54, 1046-1055.

Choi, C. H., Lee, J. M., Koo, B. B., Park, J. S., Kim, D. S., Kwon, J. S. and Kim, I. Y., 2010. Sex differences in the temporal lobe white matter and the corpus callosum: a diffusion tensor tractography study. Neuroreport 21, 73-77.

Whitwell, J. L., Crum, W. R., Watt, H. C. and Fox, N. C., 2001. Normalization of cerebral volumes by use of intracranial volume: implications for longitudinal quantitative MR imaging. AJNR Am J Neuroradiol 22, 1483-1489.

**4. Supplementary Figures**

Supplementary Figure 1: Forest plot for the intracranial volume meta-analysis.

Overview of all studies included in the intracranial volume meta-analysis. Intracranial volume is defined as the sum of grey matter, white matter and cerebrospinal fluid. The square indicates the effect size in mL of each study (i.e. the difference in mL volume between males and females) and the bars indicate the 95% confidence interval of each study. The studies corresponding to the effect size can be found on the left. Study IDs correspond to the study IDs in Table 1 and Supplementary Table 1. The diamond at the bottom of the figure indicates the meta-analytic effect size and its variance.

Supplementary Figure 2: Forest plot for the total brain volume meta-analysis.

Overview of all studies included in the total brain volume meta-analysis. Total brain volume is defined as the sum of grey matter, white matter and cerebellum. The square indicates the effect size in mL of each study (i.e. the difference in mL volume between males and females) and the bars indicate the 95% confidence interval of each study. The studies corresponding to the effect size can be found on the left. Study IDs correspond to the study IDs in Table 1 and Supplementary Table 1. The diamond at the bottom of the figure indicates the meta-analytic effect size and its variance.

Supplementary Figure 3: Forest plot for the cerebrum volume meta-analysis.

Overview of all studies included in the cerebrum volume meta-analysis. Cerebrum volume is defined as the sum of grey and white matter. The square indicates the effect size in mL of each study (i.e. the difference in mL volume between males and females) and the bars indicate the 95% confidence interval of each study. The studies corresponding to the effect size can be found on the left. Study IDs correspond to the study IDs in Table 1 and Supplementary Table 1. The diamond at the bottom of the figure indicates the meta-analytic effect size and its variance.

Supplementary Figure 4: Forest plot for the white matter volume meta-analysis.

Overview of all studies included in the white matter volume meta-analysis. The square indicates the effect size in mL of each study (i.e. the difference in mL volume between males and females) and the bars indicate the 95% confidence interval of each study. The studies corresponding to the effect size can be found on the left. Study IDs correspond to the study IDs in Table 1 and Supplementary Table 1. The diamond at the bottom of the figure indicates the meta-analytic effect size and its variance.

Supplementary Figure 5: Forest plot for the cerebrospinal fluid volume meta-analysis.

Overview of all studies included in the cerebrospinal fluid volume meta-analysis. The square indicates the effect size in mL of each study (i.e. the difference in mL volume between males and females) and the bars indicate the 95% confidence interval of each study. The studies corresponding to the effect size can be found on the left. Study IDs correspond to the study IDs in Table 1 and Supplementary Table 1. The diamond at the bottom of the figure indicates the meta-analytic effect size and its variance.

Supplementary Figure 6: Forest plot for the cerebellum volume meta-analysis.

Overview of all studies included in the cerebellum volume meta-analysis. The square indicates the effect size in mL of each study (i.e. the difference in mL volume between males and females) and the bars indicate the 95% confidence interval of each study. The studies corresponding to the effect size can be found on the left. Study IDs correspond to the study IDs in Table 1 and Supplementary Table 1. The diamond at the bottom of the figure indicates the meta-analytic effect size and its variance.

Supplementary Figure 7: Funnel plot for the intracranial volume meta-analysis.

A funnel plot measures publication bias and heterogeneity between all studies included in the intracranial volume meta-analysis. A symmetric funnel plot implies a 'well-behaving' dataset, and an asymmetric funnel plot indicates a relationship between effect-size and study size, which may be due publication bias or small-study effects. This funnel plot is symmetric (see supplementary methods).

Supplementary Figure 8: Funnel plot for the total brain volume meta-analysis.

A funnel plot measures publication bias and heterogeneity between all studies included in the total brain volume meta-analysis. A symmetric funnel plot implies a 'well-behaving' dataset, and an asymmetric funnel plot indicates a relationship between effect-size and study size, which may be due publication bias or small-study effects. This funnel plot is asymmetric and indicates publication bias and/or heterogeneity in the sample.

Supplementary Figure 9: Funnel plot for the cerebrum volume meta-analysis.

A funnel plot measures publication bias and heterogeneity between all studies included in the cerebrum volume meta-analysis. A symmetric funnel plot implies a 'well-behaving' dataset, and an asymmetric funnel plot indicates a relationship between effect-size and study size, which may be due publication bias or small-study effects. This funnel plot is symmetric.

Supplementary Figure 10: Funnel plot for the grey matter volume meta-analysis.

A funnel plot measures publication bias and heterogeneity between all studies included in the grey matter volume meta-analysis. A symmetric funnel plot implies a 'well-behaving' dataset, and an asymmetric funnel plot indicates a relationship between effect-size and study size, which may be due publication bias or small-study effects. This funnel plot is symmetric.

Supplementary Figure 11: Funnel plot for the white matter volume meta-analysis.

A funnel plot measures publication bias and heterogeneity between all studies included in the white matter volume meta-analysis. A symmetric funnel plot implies a 'well-behaving' dataset, and an asymmetric funnel plot indicates a relationship between effect-size and study size, which may be due publication bias or small-study effects. This funnel plot is symmetric.

Supplementary Figure 12: Funnel plot for the cerebrospinal fluid volume meta-analysis.

A funnel plot measures publication bias and heterogeneity between all studies included in the cerebrospinal fluid volume meta-analysis. A symmetric funnel plot implies a 'well-behaving' dataset, and an asymmetric funnel plot indicates a relationship between effect-size and study size, which may be due publication bias or small-study effects. This funnel plot is symmetric.

Supplementary Figure 13: Funnel plot for the cerebellum volume meta-analysis.

A funnel plot measures publication bias and heterogeneity between all studies included in the cerebellum volume meta-analysis. A symmetric funnel plot implies a 'well-behaving' dataset, and an asymmetric funnel plot indicates a relationship between effect-size and study size, which may be due publication bias or small-study effects. This funnel plot is symmetric.

Supplementary Figure 14: Breakdown by age categories for reports providing total brain volume.

Three plots display the breakdown of studies examining total brain volume (TBV) in the current literature across six age categories: ‘infant’ (0-1 years), ‘early childhood’ (2-6 years), ‘late childhood’ (7-17 years), ‘mature’ (18-59 years), ‘senior’ (60+ years), and ‘lifespan’ (any study covering more than 2 age ranges): (a) the total number of articles providing TBV in each age category; (b) gives the sum of the total number of male and female participants included in those age categories; and (c) displays the weighted mean volumes of TBV and weighted error bars for males and females across all age categories.

Supplementary Figure 15: Breakdown by age categories for reports providing cerebrum volume.

Three plots display the breakdown of studies examining cerebrum (Cb) volume in the current literature across six age categories: ‘infant’ (0-1 years), ‘early childhood’ (2-6 years), ‘late childhood’ (7-17 years), ‘mature’ (18-59 years), ‘senior’ (60+ years), and ‘lifespan’ (any study covering more than 2 age ranges): (a) the total number of articles providing Cb in each age category; (b) gives the sum of the total number of male and female participants included in those age categories; and (c) displays the weighted mean volumes of Cb and weighted error bars for males and females across all age categories.

Supplementary Figure 16: Breakdown by age categories for reports providing white matter volume.

Three plots display the breakdown of studies examining white matter (WM) volume in the current literature across six age categories: ‘infant’ (0-1 years), ‘early childhood’ (2-6 years), ‘late childhood’ (7-17 years), ‘mature’ (18-59 years), ‘senior’ (60+ years), and ‘lifespan’ (any study covering more than 2 age ranges): (a) the total number of articles providing WM in each age category; (b) gives the sum of the total number of male and female participants included in those age categories; and (c) displays the weighted mean volumes of WM and weighted error bars for males and females across all age categories.

Supplementary Figure 17: Breakdown by age categories for reports providing cerebrospinal fluid volume.

Three plots display the breakdown of studies examining cerebrospinal fluid (CSF) volume in the current literature across six age categories: ‘infant’ (0-1 years), ‘early childhood’ (2-6 years), ‘late childhood’ (7-17 years), ‘mature’ (18-59 years), ‘senior’ (60+ years), and ‘lifespan’ (any study covering more than 2 age ranges): (a) the total number of articles providing CSF in each age category; (b) gives the sum of the total number of male and female participants included in those age categories; and (c) displays the weighted mean volumes of CSF and weighted error bars for males and females across all age categories.

Supplementary Figure 18: Breakdown by age categories for reports providing cerebellum volume.

Three plots display the breakdown of studies examining cerebrospinal fluid (Cbl) volume in the current literature across six age categories: ‘infant’ (0-1 years), ‘early childhood’ (2-6 years), ‘late childhood’ (7-17 years), ‘mature’ (18-59 years), ‘senior’ (60+ years), and ‘lifespan’ (any study covering more than 2 age ranges): (a) the total number of articles providing Cbl in each age category; (b) gives the sum of the total number of male and female participants included in those age categories; and (c) displays the weighted mean volumes of Cbl and weighted error bars for males and females across all age categories.

Supplementary Figure 19: Funnel plot for the intracranial volume meta-analysis without two studies with large standard errors.

This funnel plot measures publication bias and heterogeneity between the studies included in the intracranial volume meta-analysis except two studies with large standard deviations (and thus large standard errors). Compared to Supplementary Figure 7, the original intracranial volume funnel plot, this plot has a more symmetric funnel shape, indicating that individual variability between studies can influence the shape of the funnel plot greatly.

**5. Supplementary Tables**

Supplementary Table 1: Articles included in the global volumes meta-analyses: Imaging and analysis parameters

Complete list of imaging and analysis parameters, and which volumes they report of all studies included in the total volumes analyses. Study IDs correspond to study IDs in the forest and funnel plots (Figure 2 and Supplementary Figures 7-13).

Supplementary Table 2: Articles included in the voxel-based volume meta-analysis: Imaging and analysis parameters

Imaging and analysis parameters of all articles that perform a voxel-based analysis of sex differences in human brain volume. Articles doing grey and/or white matter volume analyses are included in this table.

Supplementary Table 3: Articles included in the voxel-based tissue density meta-analysis: Imaging and analysis parameters

Imaging and analysis parameters of all articles that perform a voxel-based analysis of sex differences in human brain tissue density. Articles doing grey and/or white matter concentration analyses are included in this table.
