# Supplementary figures and images for: A meta-analysis of sex differences in human brain structure
Source: Neurosci Biobehav Rev. 2014 Feb;39(100):34–50. doi: 10.1016/j.neubiorev.2013.12.004 (PMC3969295; doi:10.1016/j.neubiorev.2013.12.004)

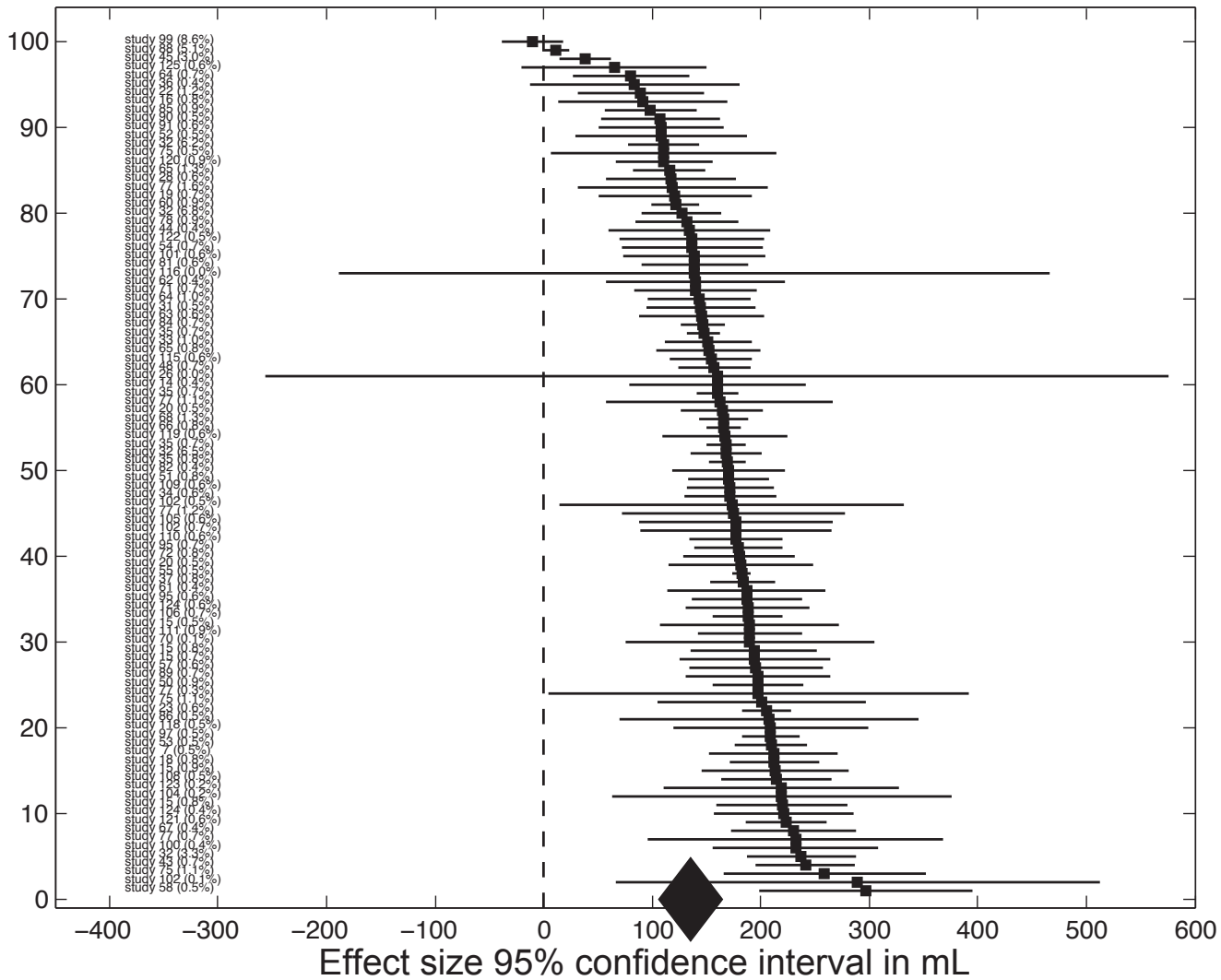

Supplement: Supplementary file 5 [file mmc5.pdf]

Study index (% variance explained)

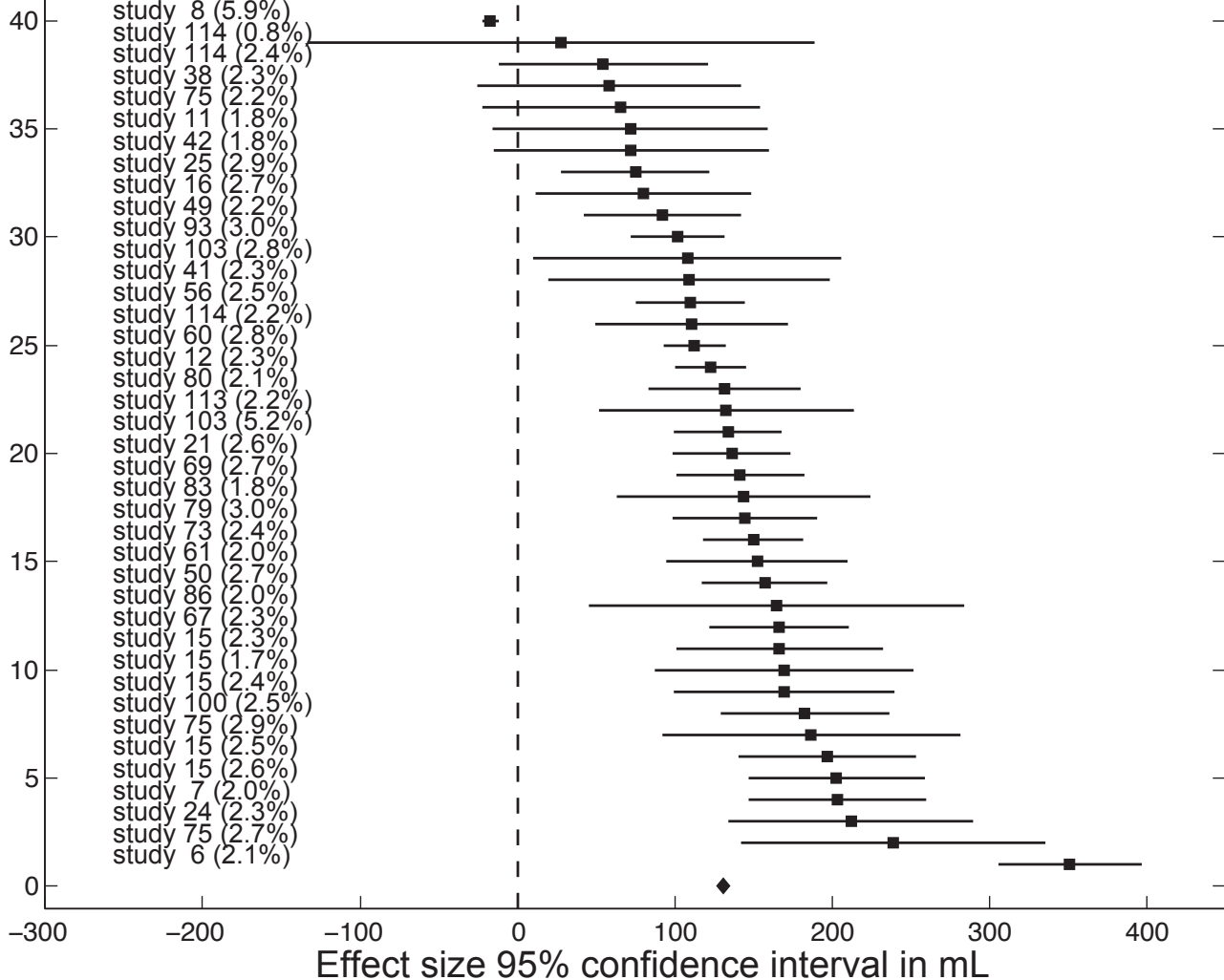

Supplement: Supplementary file 6 [file mmc6.pdf]

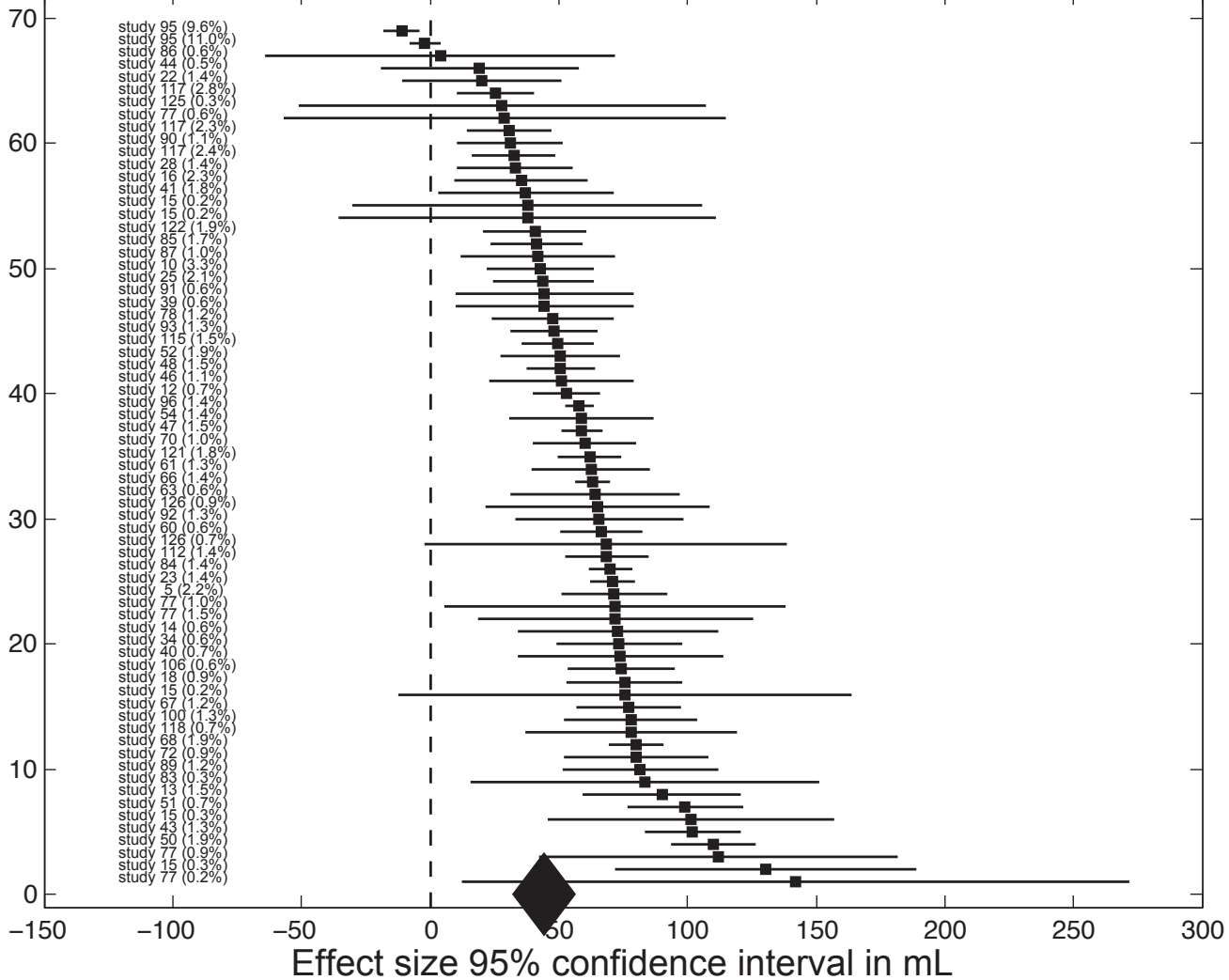

Supplement: Supplementary file 8 [file mmc8.pdf]

Study index (% variance explained)

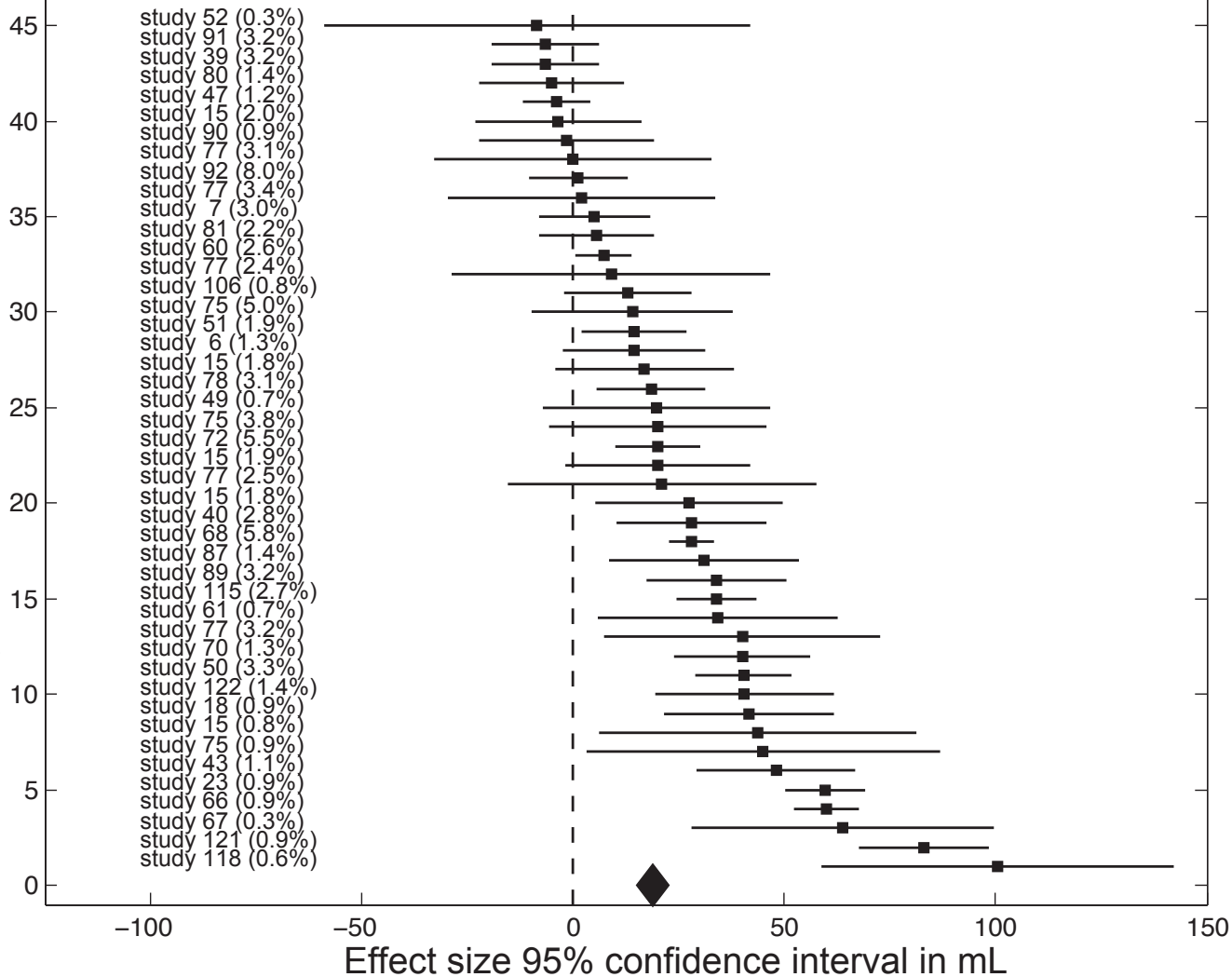

Supplement: Supplementary file 9 [file mmc9.pdf]

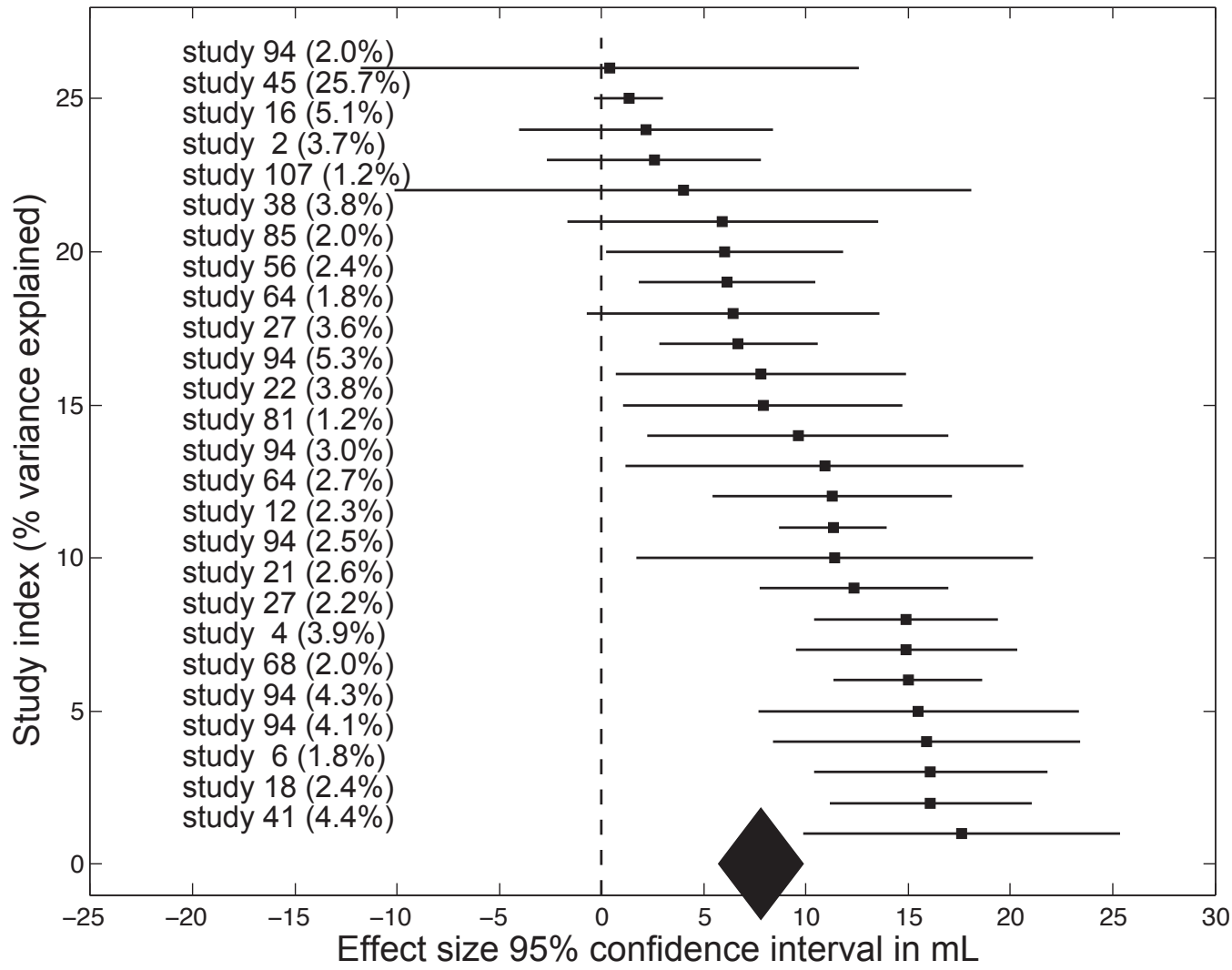

Supplement: Supplementary file 10 [file mmc10.pdf]

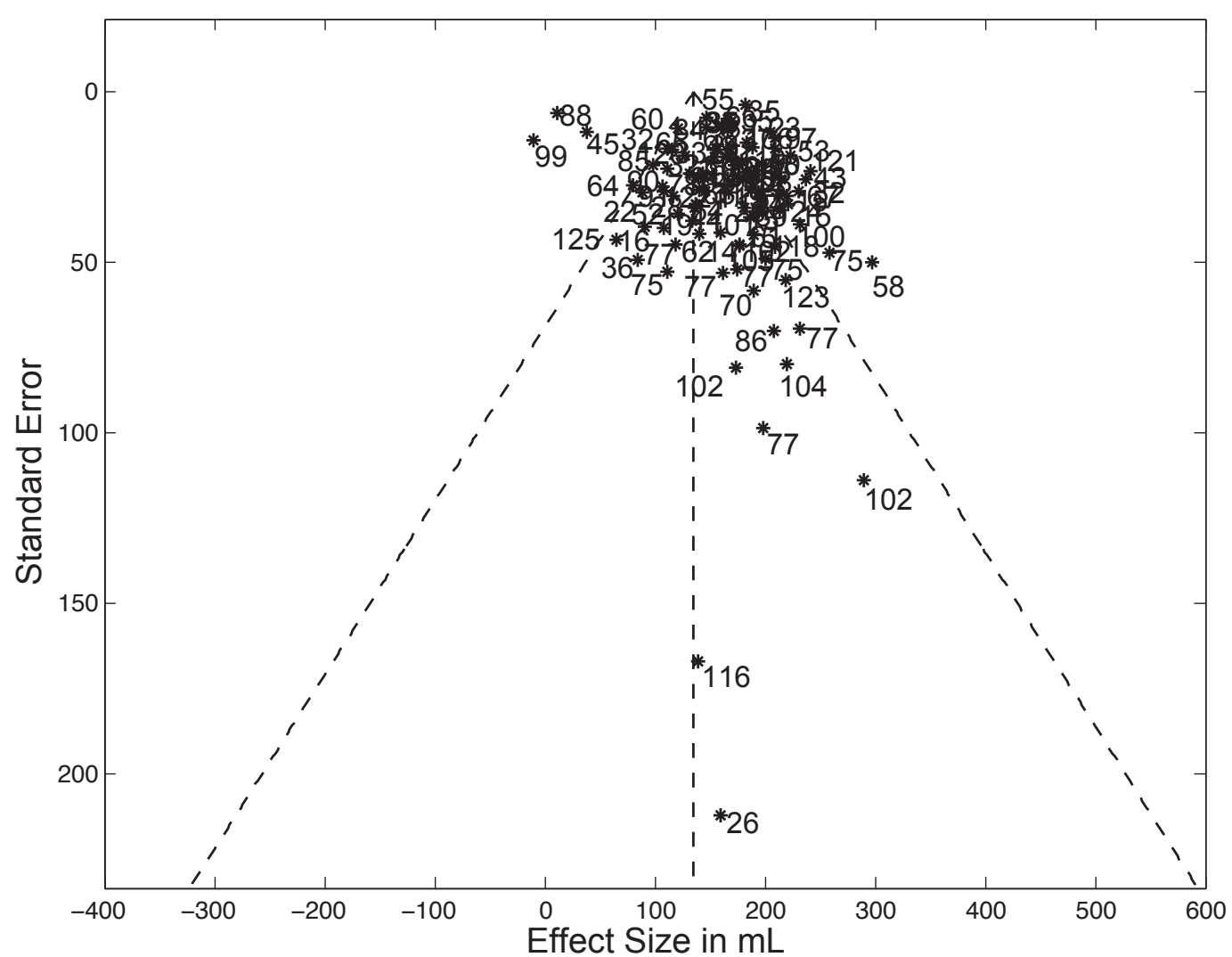

Supplement: Supplementary file 11 [file mmc11.pdf]

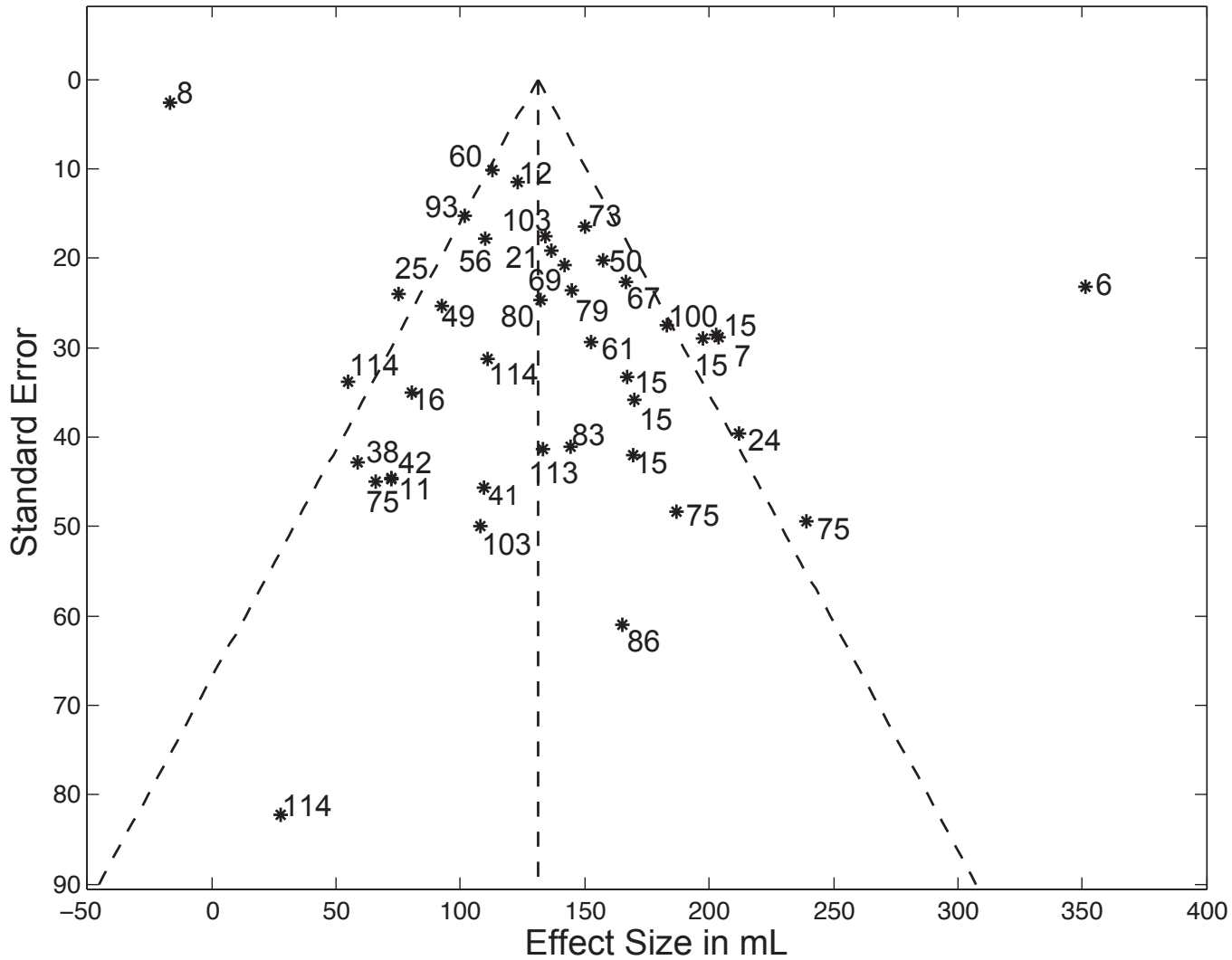

Supplement: Supplementary file 12 [file mmc12.pdf]

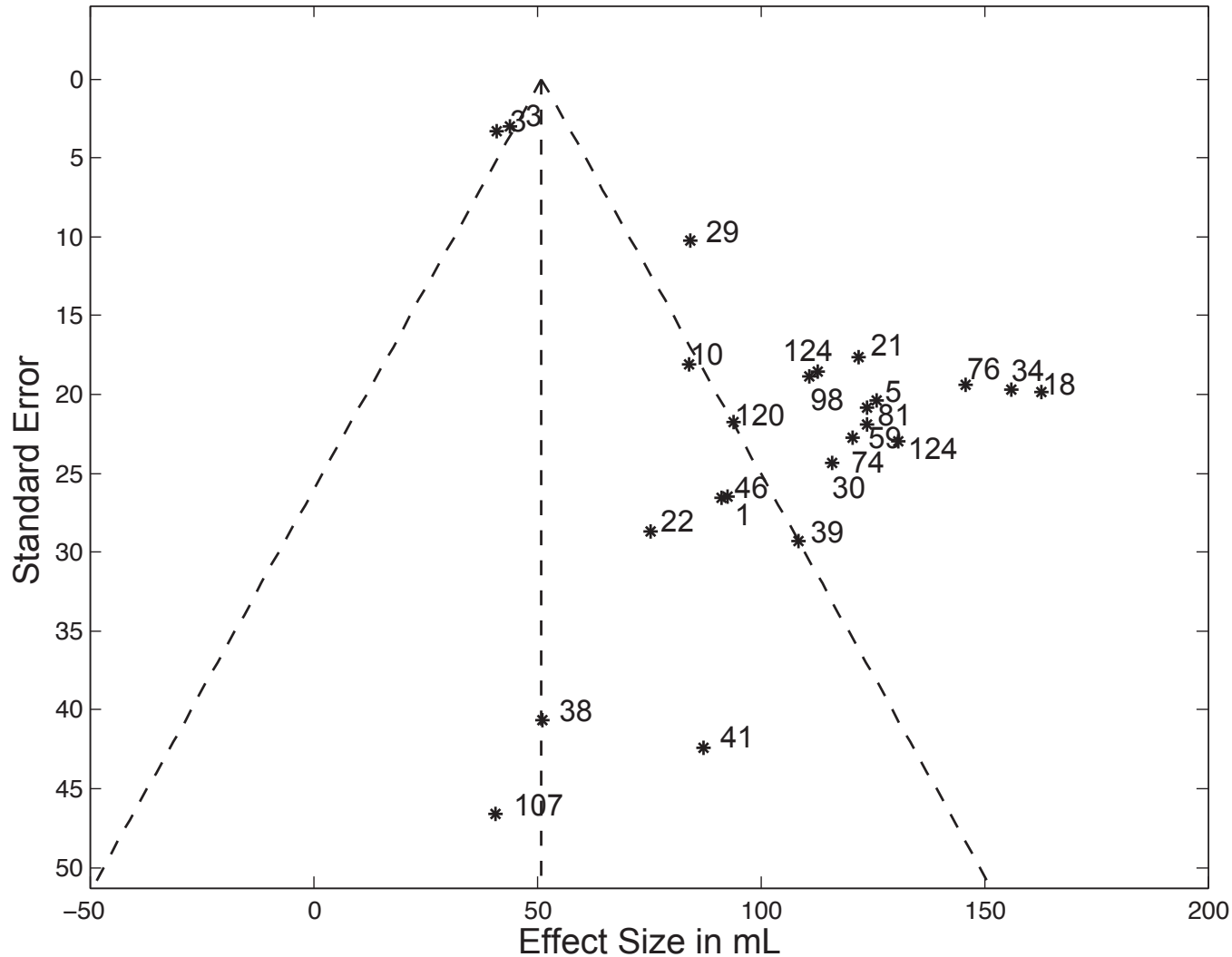

Supplement: Supplementary file 13 [file mmc13.pdf]

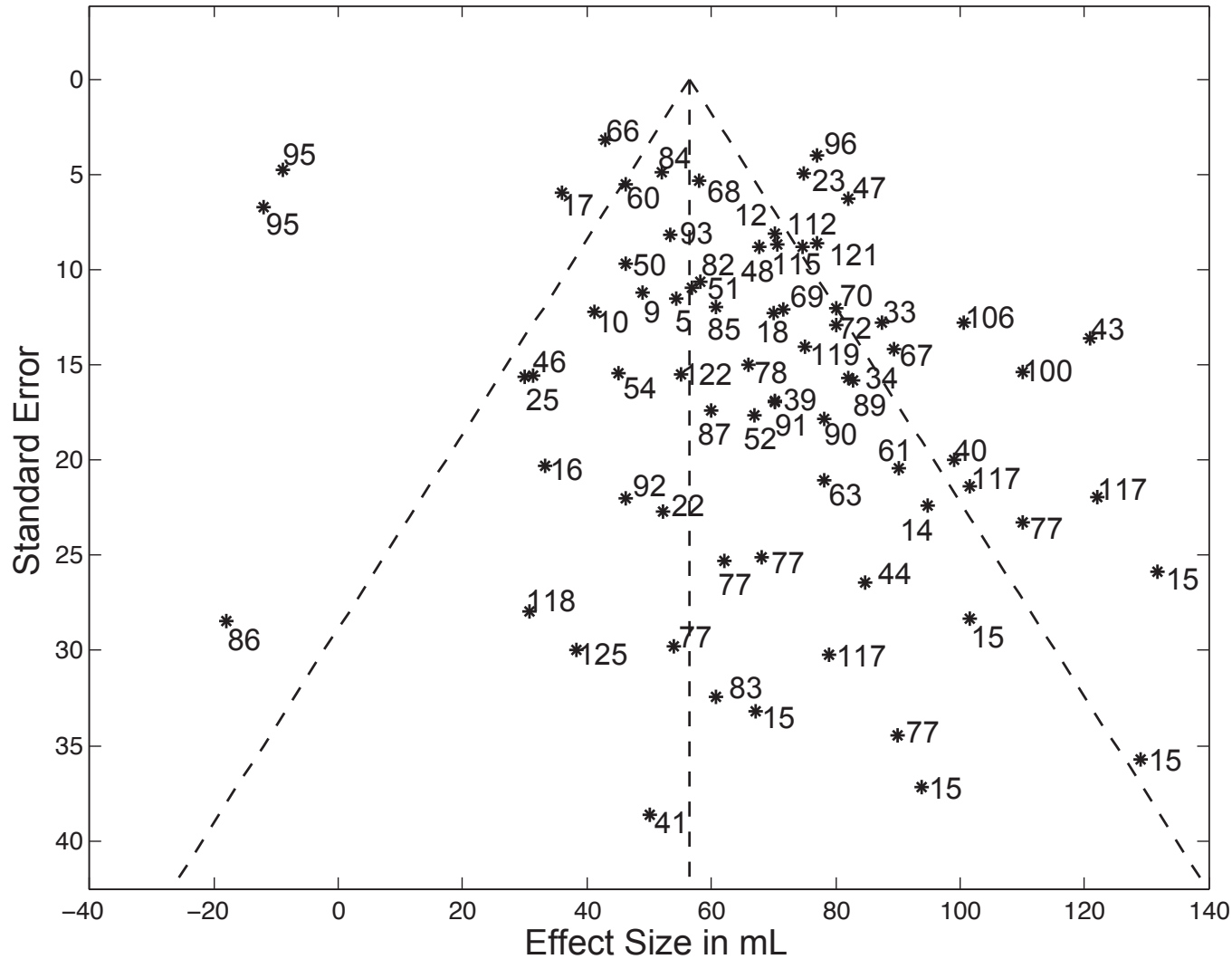

Supplement: Supplementary file 14 [file mmc14.pdf]

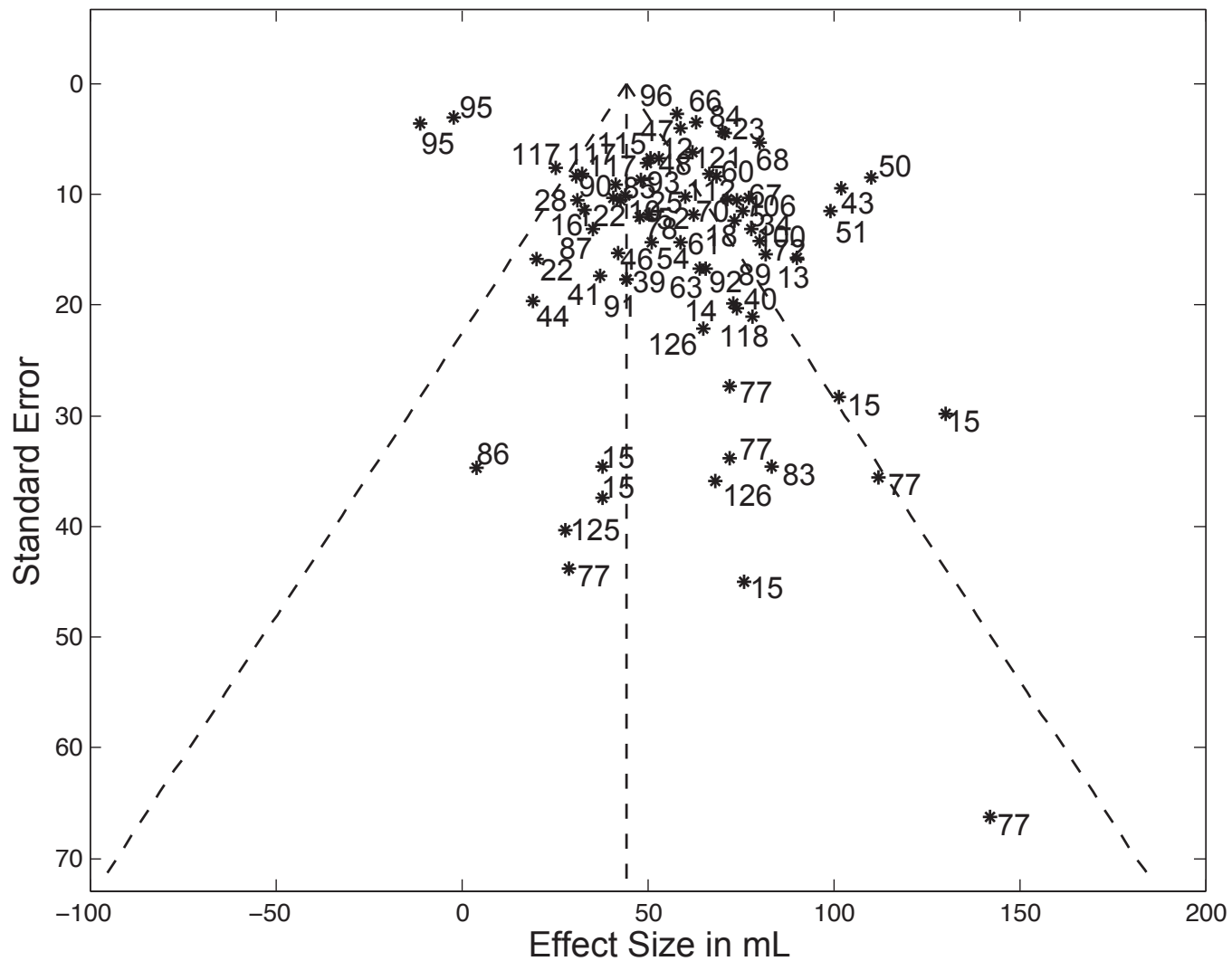

Supplement: Supplementary file 15 [file mmc15.pdf]

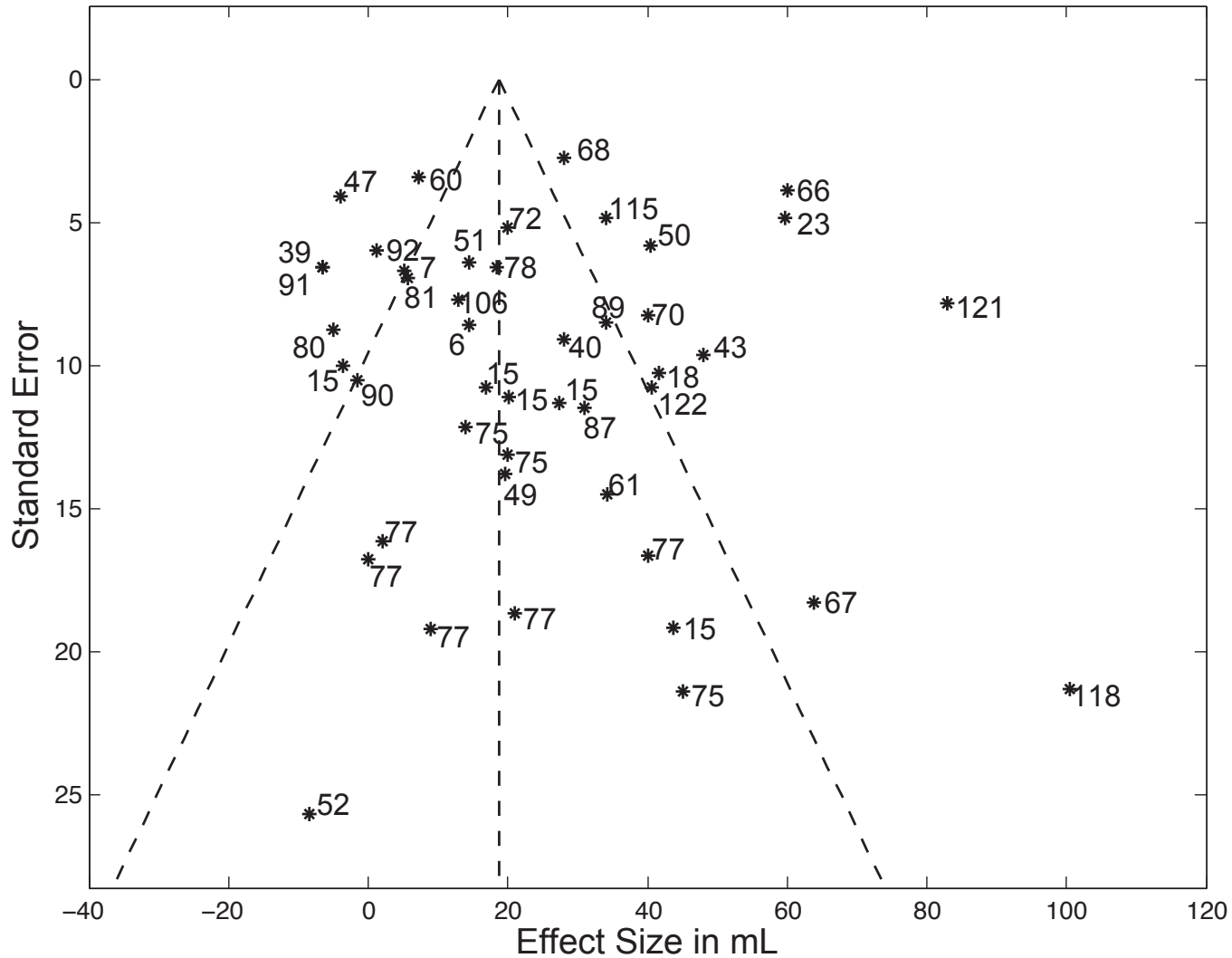

Supplement: Supplementary file 16 [file mmc16.pdf]

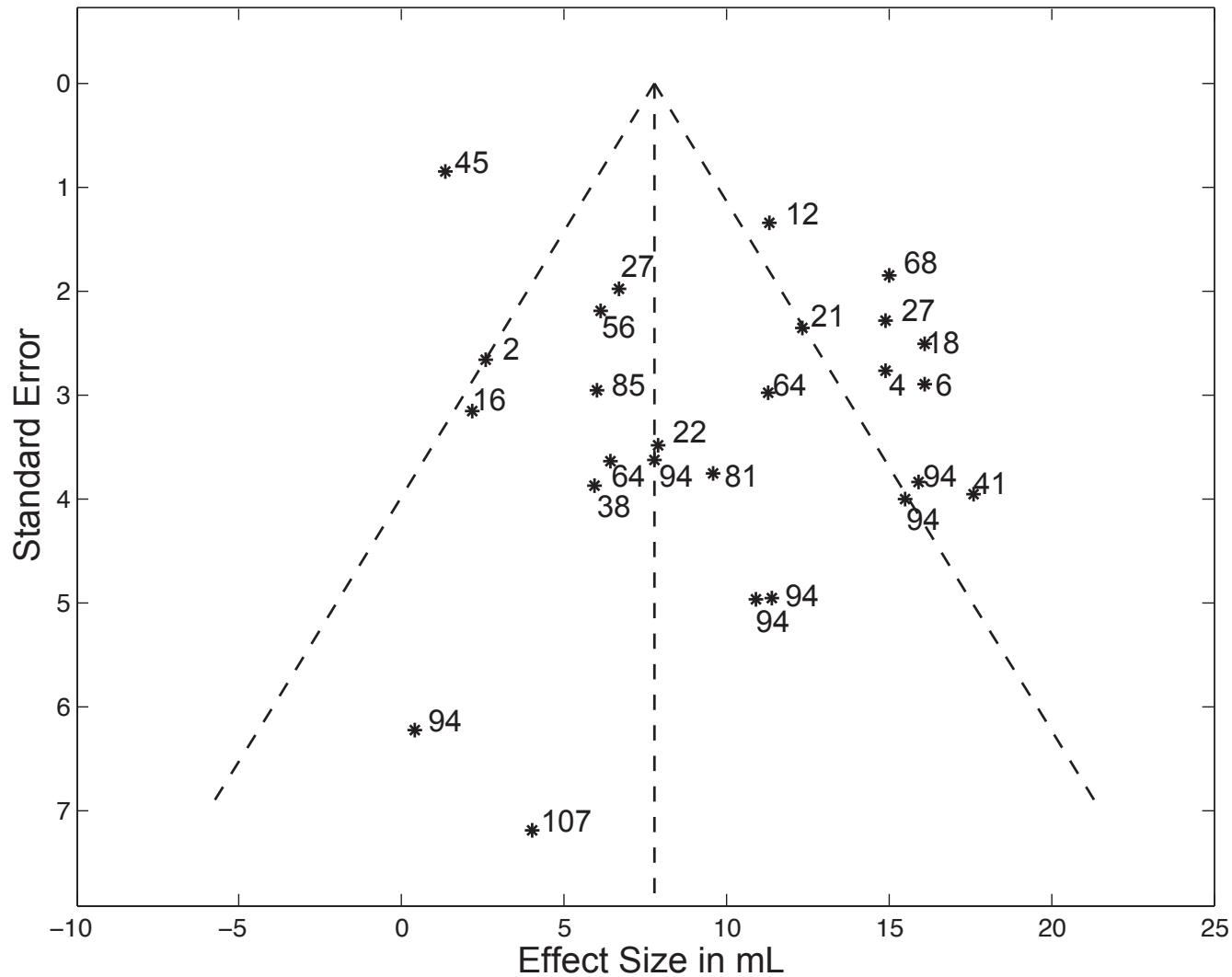

Supplement: Supplementary file 17 [file mmc17.pdf]

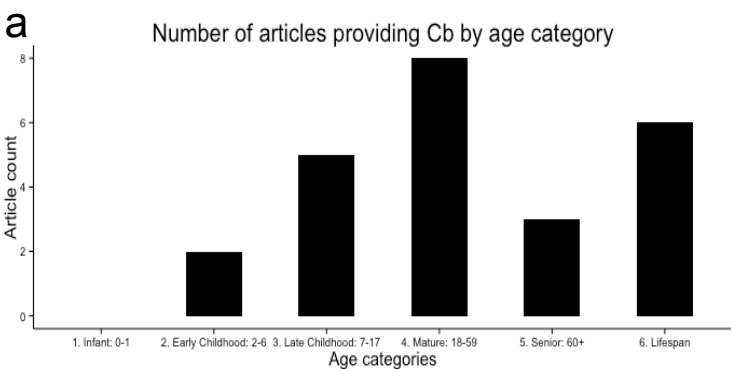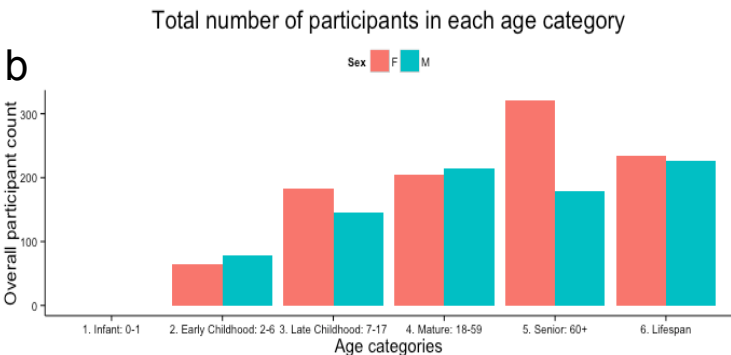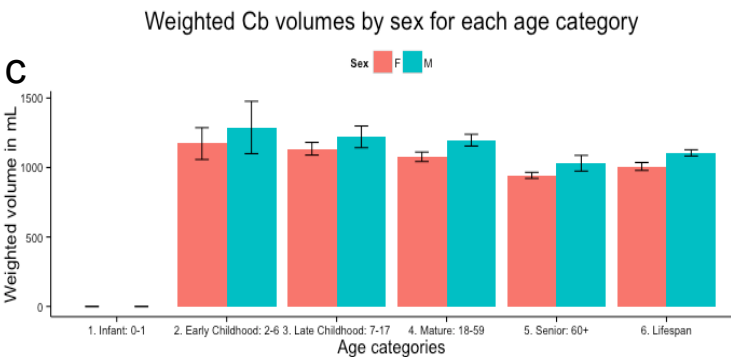

Supplement: Supplementary file 19 [file mmc19.pdf]

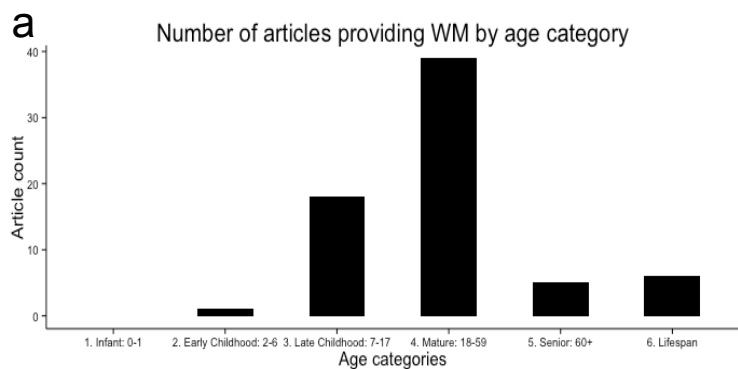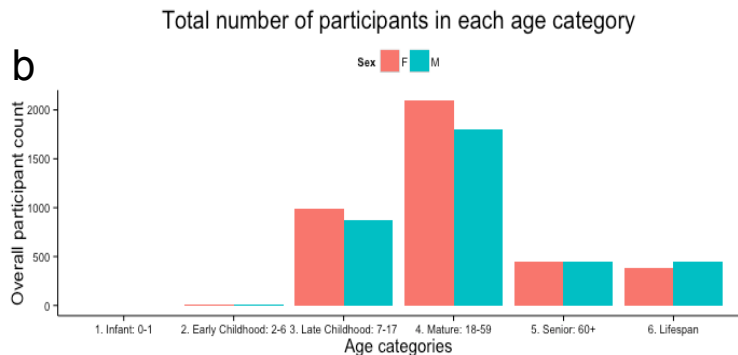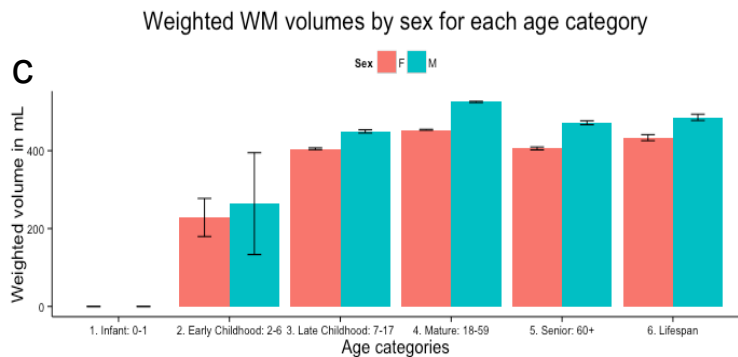

Supplement: Supplementary file 20 [file mmc20.pdf]

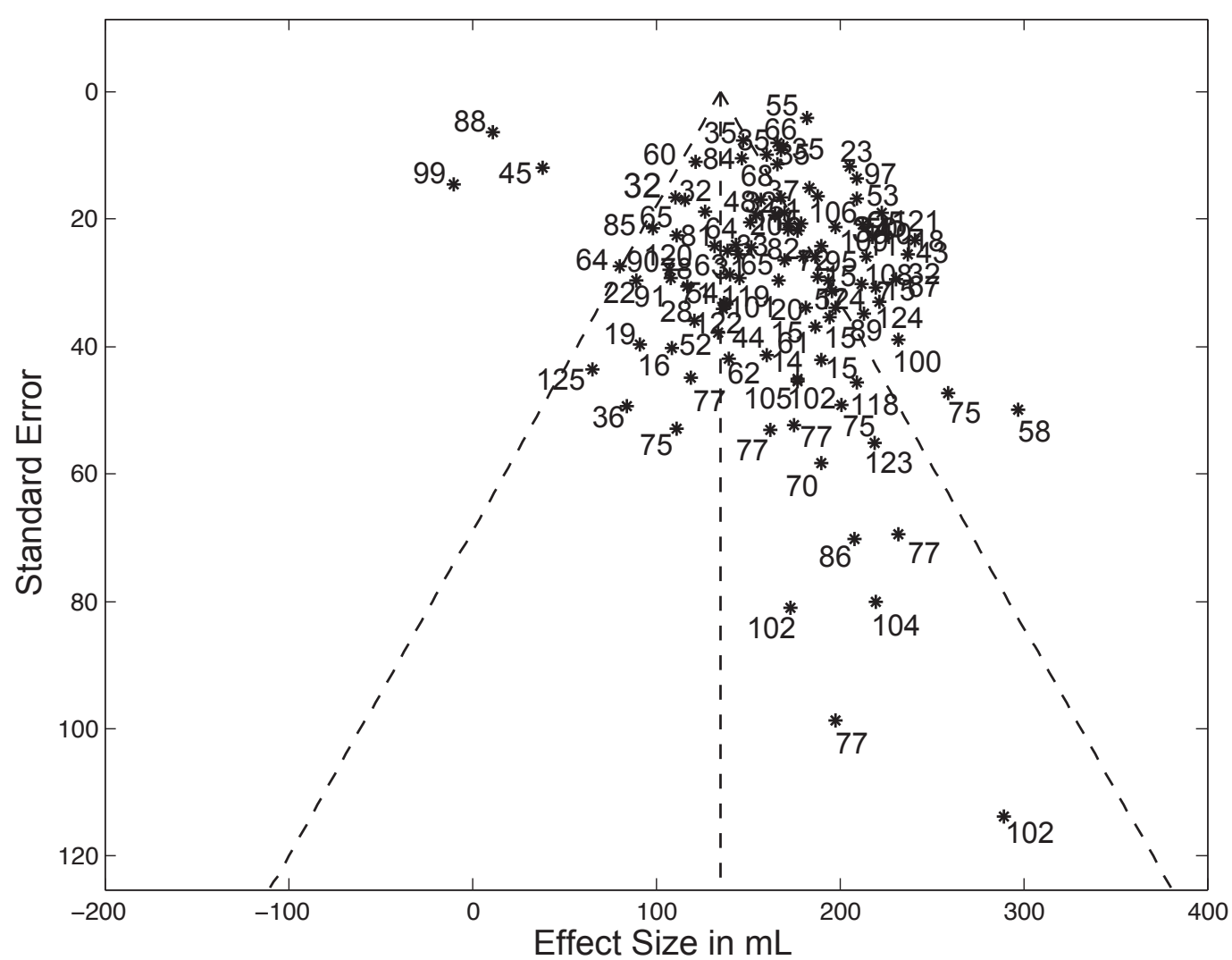

Supplement: Supplementary file 23 [file mmc23.pdf]
