# Supplementary material for: A meta-analysis of sex differences in human brain structure
Source: Neurosci Biobehav Rev. 2014 Feb;39(100):34–50. doi: 10.1016/j.neubiorev.2013.12.004 (PMC3969295; doi:10.1016/j.neubiorev.2013.12.004)
Supplement: Supplementary file 7 [file mmc7.pdf]

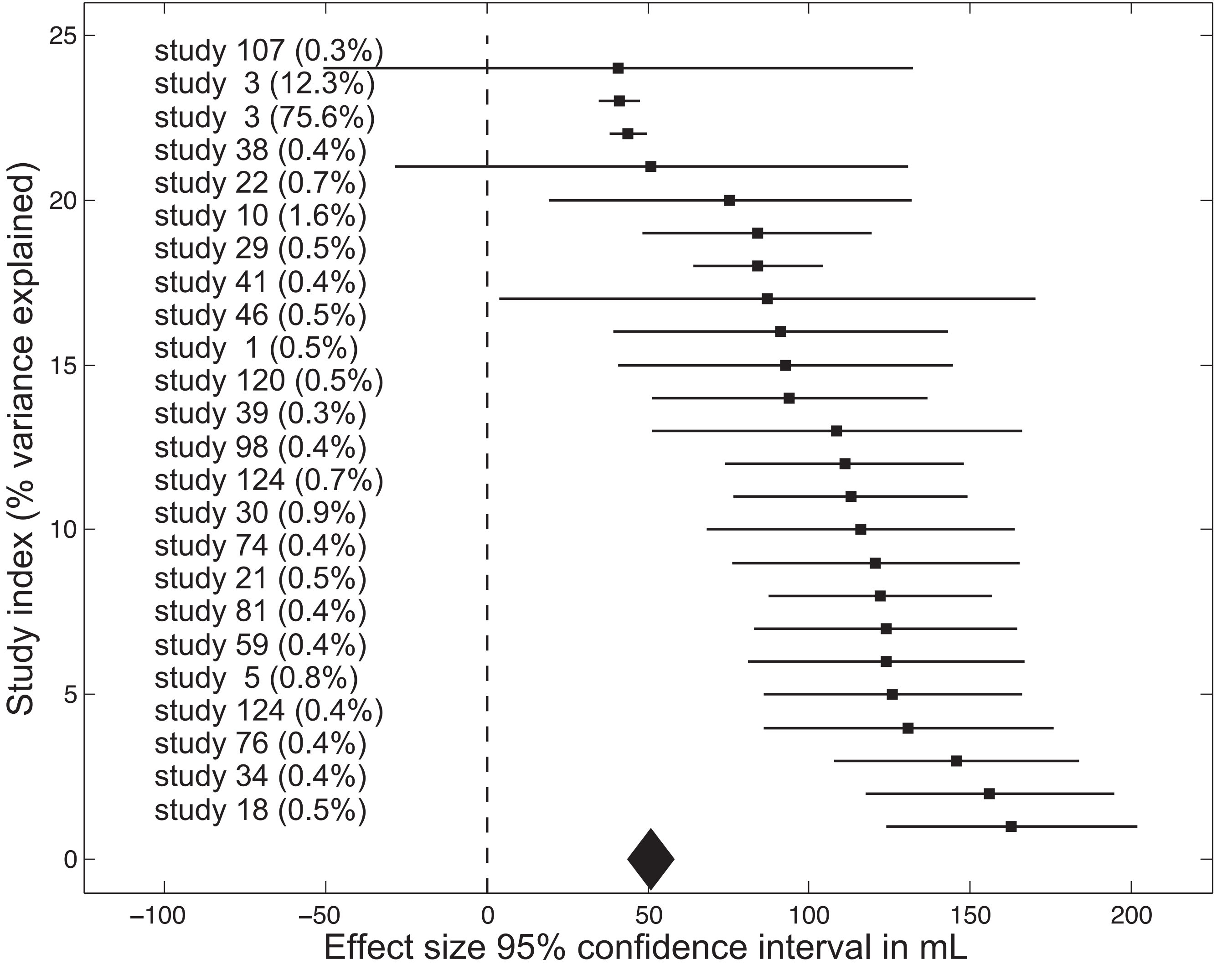
study 107 (0.3%)
study 3 (12.3%)
study 3 (75.6%)
study 38 (0.4%)
study 22 (0.7%)
study 10 (1.6%)
study 29 (0.5%)
study 41 (0.4%)
study 46 (0.5%)
study 1 (0.5%)
study 120 (0.5%)
study 39 (0.3%)
study 98 (0.4%)
study 124 (0.7%)
study 30 (0.9%)
study 74 (0.4%)
study 21 (0.5%)
study 81 (0.4%)
study 59 (0.4%)
study 5 (0.8%)
study 124 (0.4%)
study 76 (0.4%)
study 34 (0.4%)
study 18 (0.5%)
Study index (% variance explained)
25
20
15
10
5
0
−100
−50
0
50
100
150
200
Effect size 95% confidence interval in mL
