# Supplementary material for: A meta-analysis of sex differences in human brain structure
Source: Neurosci Biobehav Rev. 2014 Feb;39(100):34–50. doi: 10.1016/j.neubiorev.2013.12.004 (PMC3969295; doi:10.1016/j.neubiorev.2013.12.004)
Supplement: Supplementary file 18 [file mmc18.pdf]

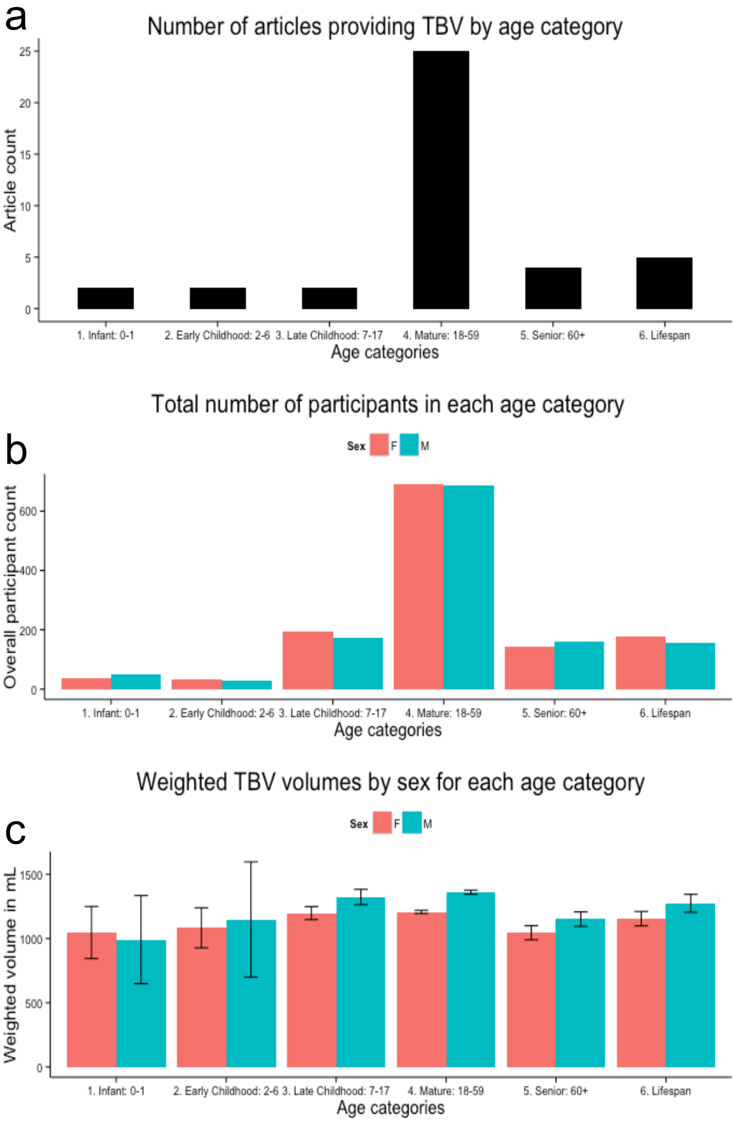
a
Number of articles providing TBV by age category
Article count
0
5
10
15
20
25
1. Infant: 0-1
2. Early Childhood: 2-6
3. Late Childhood: 7-17
4. Mature: 18-59
5. Senior: 60+
6. Lifespan
Age categories
Total number of participants in each age category
b
Sex
F
M
Overall participant count
0
200
400
600
1. Infant: 0-1
2. Early Childhood: 2-6
3. Late Childhood: 7-17
4. Mature: 18-59
5. Senior: 60+
6. Lifespan
Age categories
Weighted TBV volumes by sex for each age category
c
Sex
F
M
Weighted volume in mL
0
500
1000
1500
1. Infant: 0-1
2. Early Childhood: 2-6
3. Late Childhood: 7-17
4. Mature: 18-59
5. Senior: 60+
6. Lifespan
Age categories
